# Smash++: an alignment-free and memory-efficient tool to find genomic rearrangements

**DOI:** 10.1093/gigascience/giaa048

**Published:** 2020-05-20

**Authors:** Morteza Hosseini, Diogo Pratas, Burkhard Morgenstern, Armando J Pinho

**Affiliations:** 1 IEETA/DETI, University of Aveiro, Campus Universitário de Santiago, 3810-193 Aveiro, Portugal; 2 Department of Virology, University of Helsinki, Haartmaninkatu 3, 00014 Helsinki, Finland; 3 Department of Bioinformatics, University of Göttingen, Goldschmidtstr. 1, 37077 Göttingen, Germany; 4 Göttingen Center of Molecular Biosciences (GZMB), Justus-von-Liebig-Weg 11, 37077 Göttingen, Germany

**Keywords:** genomic rearrangement, alignment-free, genome comparison, genome duplication, data compression, information theory, probabilistic-algorithmic model, complexity, visualization, high-throughput sequencing

## Abstract

**Background:**

The development of high-throughput sequencing technologies and, as its result, the production of huge volumes of genomic data, has accelerated biological and medical research and discovery. Study on genomic rearrangements is crucial owing to their role in chromosomal evolution, genetic disorders, and cancer.

**Results:**

We present Smash++, an alignment-free and memory-efficient tool to find and visualize small- and large-scale genomic rearrangements between 2 DNA sequences. This computational solution extracts information contents of the 2 sequences, exploiting a data compression technique to find rearrangements. We also present Smash++ visualizer, a tool that allows the visualization of the detected rearrangements along with their self- and relative complexity, by generating an SVG (Scalable Vector Graphics) image.

**Conclusions:**

Tested on several synthetic and real DNA sequences from bacteria, fungi, Aves, and Mammalia, the proposed tool was able to accurately find genomic rearrangements. The detected regions were in accordance with previous studies, which took alignment-based approaches or performed FISH (fluorescence *in situ* hybridization) analysis. The maximum peak memory usage among all experiments was ∼1 GB, which makes Smash++ feasible to run on present-day standard computers.

## Background

With the ever-increasing development of high-throughput sequencing (HTS) technologies, a massive amount of genomic information is produced at much higher speed and lower cost than was possible before [1]. Analysis of such information has led to the advancement of our understanding of biology and disease over the past decade [2, 3]. Computational solutions play a key role in dry-lab analysis of the deluge of HTS data by using efficient and fast algorithms.

Genome rearrangements are mutations that alter the arrangement of genes on a genome, and they usually occur in the presence of errors in cell division following meiosis or mitosis. These structural abnormalities in chromosomes, wich include but are not limited to deletions, duplications, translocations, inversions, and insertions, mostly occur as an accident in the sperm or egg cell and hence are present in every cell of the body [4, 5].

Studies on chromosomal aberrations, which underlie many genetic diseases and cancer, are crucial for diagnostics, prognostics, and targeted therapeutics [6, 7]. Examples of such diseases are Wolf-Hirschhorn syndrome, which is caused by a partial deletion from human chromosome location 4p16.3 [8]; Charcot-Marie-Tooth disease, which is most commonly caused by duplication of the gene encoding peripheral myelin protein 22 on human chromosome 17 [9]; and acute myeloid leukemia, which may be caused by translocations between human chromosomes 8 and 21 [10].

Various computational methods have been proposed in the literature that perform alignment, i.e., aligning regions that are conserved in 2 (or more) genomic sequences, for the purpose of detecting chromosomal rearrangements (comparing sequences) [11–16]. Alignment methods cannot be solely used to detect rearrangements because they follow the assumption that order of homology is maintained between the sequences to be compared [17, 18]. Alignment-free (AF) approaches, on the other hand, do not have this limitation; in addition, they offer computational speed-up advantages over alignment algorithms [19].

Among AF methods are information theory–based ones, which measure the amount of shared information within the sequences to quantify the similarity/dissimilarity between them. Information theory–based approaches have a broad range of applications, including but not limited to global and local characterization of DNA, e.g., prediction of transcription factor binding sites and classification of motifs, and gene mapping [20]. In 2015 another application of such approaches, namely, finding rearrangements between DNA sequences, was introduced [21]. Here, we provide a significant improvement over the mentioned method. It should be noted that the 2 alignment-based and AF approaches can be accompanied by genomic data visualization tools, which provide researchers with facilities to explore and analyze genomic data [22].

In this article we present Smash++, an AF tool that finds chromosomal rearrangements between 2 DNA sequences based on their information content, which is obtained by a data compression technique. This computational solution follows a combination of probabilistic and algorithmic approaches for having a quantitative definition of information, although it can be seen as more of a probabilistic one [23]. Associated with Smash++, we present a visualizer that is capable of visualizing as SVG images informationally similar regions between 2 genomic sequences. This tool also provides self- and relative redundancy (complexity) for the similar regions.

Smash++ is an improved version of Smash [21], featuring (i) improved accuracy, obtained by using multiple finite-context models (FCMs) along with substitution-tolerant Markov models to find fine-grained and coarse-grained chromosomal rearrangements; (ii) presentation of self-complexity (redundancy) and relative redundancy of informationally similar regions between 2 DNA sequences; (iii) improved user interface in the command line, by adding several options to customize the tool for running, and the resulting SVG image, by adding markers for positions of DNA bases and also plotting self- and relative redundancy; and (iv) improved performance, in terms of memory and time usage.

## Results and Discussion

### Implementation

Smash++ is implemented in the C++ language and is licensed under GNU GPLv3. It generates information maps for 2 sequences and, based on that, finds similar regions in them, in which there can potentially be DNA rearrangements. Therefore, Smash++ provides insight into positions of rearrangements that have happened between 2 sequences. The tool comes with a visualizer, which can be called in the command line with a flag called “-viz”. Similar regions in reference and target sequences are shown with the same color, which is chosen randomly using an HSV color model. For more information about use of the tool, see Note S3 of the supplementary material.

The machine used for the tests had a 2-core 2.90 GHz Intel^®^ Core™ i7-3520M CPU and 8 GB of RAM. The Python script “xp.py,” in the “experiment” directory, can be used to reproduce the results by switching False/True the variables associated with each dataset.

### Dataset

Smash++ and several other methods have been tested on a collection of synthetic and real sequences, which are described in Table 1. We used the GOOSE toolkit [24] to make the synthetic sequences, of which the sizes vary from 1.5 kb to 100 Mb. We applied mutations and reversely complemented parts of the sequences. For a real dataset, we chose different sequences from bacteria, Aves, Mammalia, and fungi, with sizes of ∼1 to ∼127 Mb.

**Table 1:** Synthetic and real dataset used in the experiments

Sequence	Group	Length (bp)	Description
GGA18	Aves	11,373,140	Access. CM000110 – *Gallus gallus* chromosome 18
MGA20	Aves	10,730,484	Access. CM000981 – *Meleagris gallopavo* isolate NT-WF06-2002-E0010 breed Aviagen turkey brand Nicholas breeding stock chromosome 20
GGA14	Aves	16,219,308	Access. CM000106 – *G. gallus* chromosome 14
MGA16	Aves	14,878,991	Access. CM000977 – *Meleagris gallopavo* isolate NT-WF06-2002-E0010 breed Aviagen turkey brand Nicholas breeding stock chromosome 16
HS12	Mammalia	133,275,309	Access. NC_000012 – *Homo sapiens* chromosome 12, GRCh38.p13 Primary Assembly
PT12	Mammalia	130,995,916	Access. NC_036891 – *Pan troglodytes* isolate Yerkes chimp pedigree #C0471 (Clint) chromosome 12
PXO99A	Bacteria	5,238,555	Access. CP000967 – *Xanthomonas oryzae* pv. *oryzae* causes the major disease of bacterial blight of rice (*Oryza sativa L*.). *X. oryzae* pv. *oryzae* PXO99A strain is virulent toward a large number of rice varieties representing diverse genetic sources of resistance [25]
MAFF 311018	Bacteria	4,940,217	Access. AP008229 – *X. oryzae* pv. *oryzae* MAFF 311018 is a Japanese race 1 strain [26]
ScVII	Fungi	1,090,940	Access. NC_001139 – *Saccharomyces cerevisiae* S288C chromosome VII
SpVII	Fungi	1,105,967	Access. CP020299 – *Saccharomyces paradoxus* strain UFRJ50816 chromosome VII
RefS	Synthetic	1,500	It consists of 3 segments of 500 bp size.
TarS	Synthetic	1,500	To build TarS, segment I is mutated 2%, II is inversely repeated, and III is duplicated.
RefM	Synthetic	100,000	It has 4 segments of 25 kb size.
TarM	Synthetic	100,000	For building TarM, segment I of RefM (out of total 4) is inversely repeated, II is mutated 90%, III is duplicated, and IV is mutated 3%
RefL	Synthetic	5,000,000	It includes 2 segments, 2,500,000 bp each
TarL	Synthetic	5,000,000	Segment I is inversely repeated, and II is mutated 2% for building TarL
RefXL	Synthetic	100,000,000	It is made of 4 segments, 25,000,000 bp each
TarXL	Synthetic	100,000,000	Segment I is mutated 1%, segments II and III are inversely repeated, and segment IV is duplicated to make TarXL
RefMut	Synthetic	60,000	It includes 60 segments of 1 kb size
TarMut	Synthetic	60,000	To build TarMut, the first segment (I) is mutated 1%, the second segment is mutated 2%, the third one is mutated 3%, and so on
RefComp	Synthetic	1,000,000	It consists of 10 segments of 100 kb
TarComp	Synthetic	1,000,000	To build it, the first segment (I) of RefComp is duplicated, and the second, third, and fourth segments are mutated 1%, 2%, and 3%, respectively. Segments V, VI,and VII of RefComp are inversely repeated, then mutated 4%, 5%, and 6%, respectively. Finally, segments VIII, IX, and X are mutated 7%, 8%, and 9%, respectively.
RefPerm	Synthetic	3,000,000	It includes 3 segments of 1 Mb size. In addition to the original sequence, it is permutated, using GOOSE toolkit, by blocks of sizes 450 kb, 30 kb, 1 kb and 30 bp.
TarPerm	Synthetic	3,000,000	To build TarPerm, the first segment is mutated 1%, the second segment is inversely repeated, and the third one is mutated 2%.

The real dataset can be download from NCBI via accession number (access.) provided in the descriptions.

### Application on synthetic data

**Figure 1:**
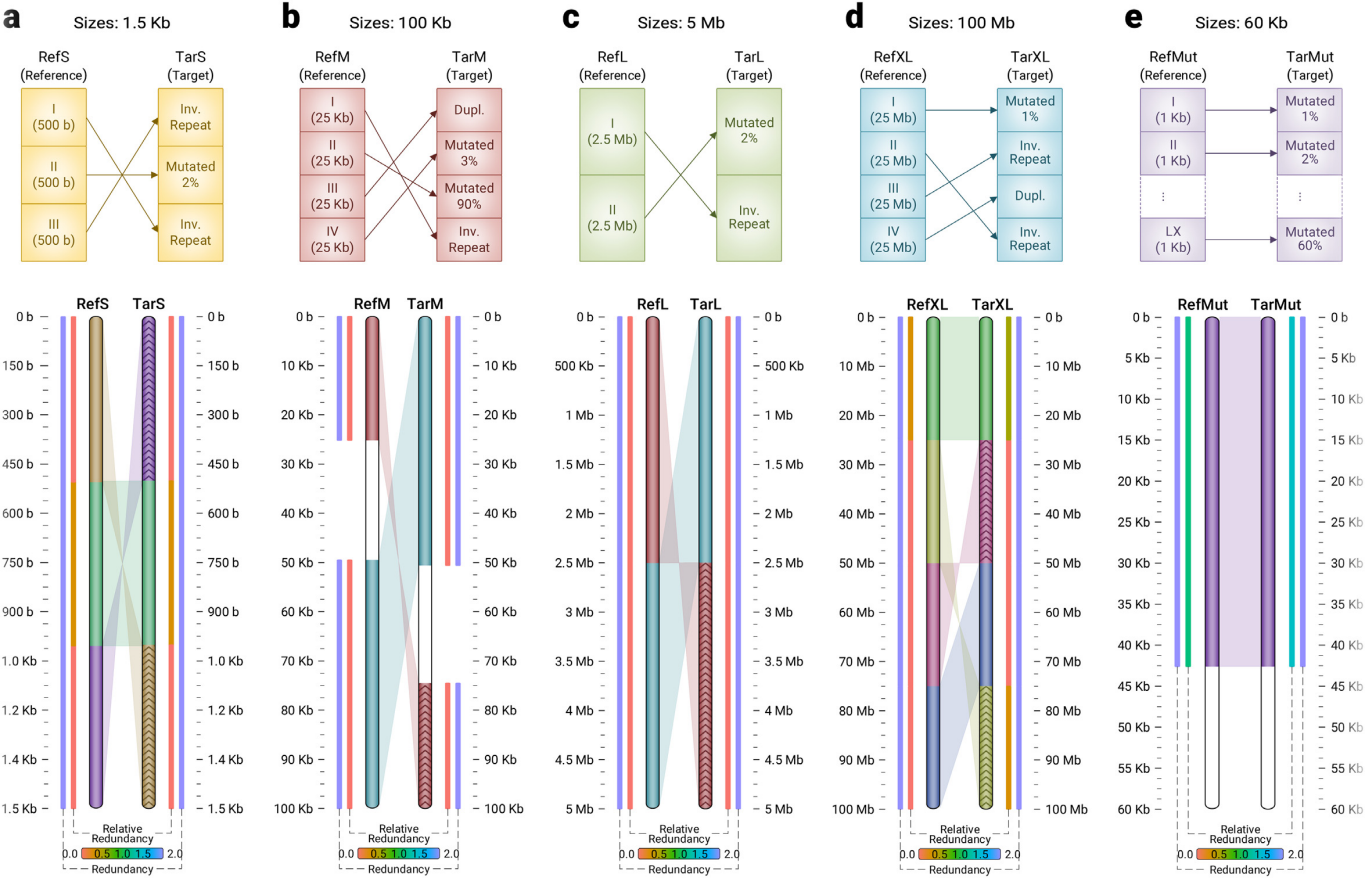
Similarities between synthetic sequences with different sizes, detected by Smash++. The parameters used are *k*-mer size = 14 and number of substitutions in substitution-tolerant Markov model (STMM) = 5, which are the default parameters used by Smash++. For the threshold, the default values of 1.5 and 1.97 are used for panels a–d and e, respectively. (a) 1.5 kb sequences; (b) 100 kb sequences. No similarity is detected for Part II of the reference because it is mutated 90%. Parts III and IV of the reference and I and II of the target are joined because there is no space between consecutive regions. (c) 5 Mb sequences; (d) 100 Mb sequences; (e) 60 kb sequences. Roughly 43% of mutation is detected.

Fig. 1 illustrates the result of running Smash++ and the associated visualizer on a synthetic dataset. The top sections show how we have built the reference and the target sequences. For example, to build the reference sequence in Fig. 1a, we generated 3 random sequences of size 500 bp, using GOOSE, and concatenated them. To build the target sequence, we made reverse complements of Parts I and III from the reference, and also mutated Part II 2%, then we concatenated the parts in the order shown in the figure. Fig. 1b, c, and d follow the same procedure. To build the target in Fig. 1e, we mutated the first 1 kb block of the reference 1%, the second block 2%, and the third block 3%, up until the 60th block, which we mutated 60%.

The bottom sections of Fig. 1 show the output of the Smash++ visualizer, detecting similar regions regardless of their size. Note that for each detected region, the average value of redundancy and relative redundancy is illustrated. In Fig. 1b, Part II of the reference is mutated 90%; i.e., 9 of every 10 bases are mutated, on average. As expected, Smash++ does not recognize similarity between this pair of regions. Also, in the case of Parts III and IV of the reference, because we detect similarity between Part III of the reference and I of the target, and also Part IV of the reference and II of the target, and there is no space between these regions, we join them and consider them as a bigger region of size 50 kb. Fig. 1e shows that Smash++ is able to detect ∼43% of mutation, which has been made possible by the use of substitution-tolerant Markov models (see Methods). Fig. 1 shows that Smash++ can be used to detect small-scale and large-scale similarities between DNA sequences.

### Application on real data

**Figure 2:**
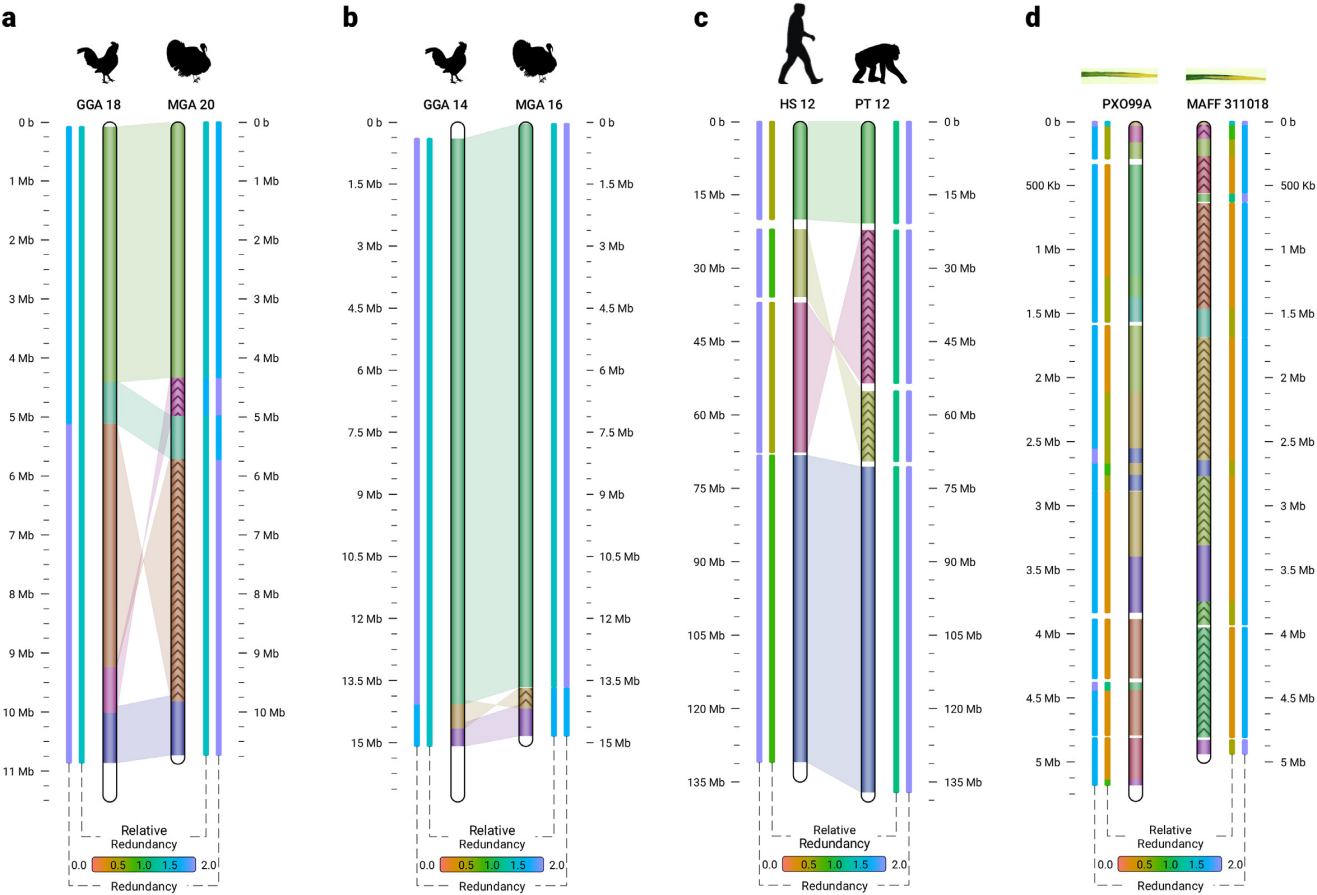
Similarities in a real dataset, detected by Smash++. (a) *G. gallus* (chicken) chr. 18 and *M. gallopavo* (turkey) chr. 20. The parameters were *k*-mer size = 14, No. substitutions in STMM = 5, threshold = 1.9, and minimum block size (*m*) = 500,000; i.e., regions smaller than 500,000 bp were not considered for further processing; (b) *G. gallus* chr. 14 and *M. gallopavo* chr. 16. The result is obtained by setting *k* = 14, No. substitutions = 5, threshold = 1.95, and *m* = 400,000; (c) *H. sapiens* (human) chr. 12 and *P. troglodytes* (chimpanzee) chr. 12. The parameters were *k* = 14, without using STMM, threshold = 1.9, and *m* = 100,000; (d) *X. oryzae* pv. *oryzae* PXO99A (a rice pathogen) and *X. oryzae* pv. *oryzae* MAFF 311018 (a rice pathogen). The result was obtained by setting *k* = 13, threshold = 1.55, and *m* = 10,000.

Fig. 2 shows similarities between real sequences, found by Smash++. Fig. 2a and b show similarities of chromosomes 18 and 14 of *Gallus gallus* (chicken) with orthologous chromosomes 20 and 16 of *Meleagris gallopavo* (turkey), respectively. These avian species, which are of great agricultural and commercial importance, are estimated to have diverged 37.2 million years ago (MYA) [27]. Fig. 2a and  b demonstrate that Smash++ was able to find the inversions confirmed by fluorescence *in situ* hybridization (FISH) analysis [28, 29].

In Figs S1 and S2 of the supplementary material, we have compared Smash++ with other methods, on GGA 18 / MGA 20 and GGA 14 / MGA 16 chromosomes, respectively. The methods included in these figures are as follows: (a) Smash++; (b) progressiveMauve [11], which uses an alignment objective score to detect rearrangement breakpoints when genomes have unequal gene content. It also applies a probabilistic alignment filtering method to remove erroneous alignments of unrelated sequences; (c) the method proposed in [29], which takes a bacterial artificial chromosome–based approach along with FISH analysis to develop an integrated physical, genetic, and comparative map of chicken and turkey; (d) SynBrowser [15], which constructs synteny blocks using prebuilt alignments in the UCSC genome browser database; and (e) FISH analysis [28].

Fig. 2c demonstrates similarities between chromosomes 12 of *Homo sapiens* and *Pan troglodytes*, which are estimated to have diverged 6.7 MYA. A comparison with other methods is provided in Fig. S3 of the supplementary material. The methods include (a) Smash++; (b) progressiveMauve; (c) Cinteny [16], which performs sensitivity analysis for synteny block detection and for the subsequent computation of reversal distances, by means of an extended version of ternary search trees. Embedded in this extension are “walks” through the leaves of the tree, which correspond to walks on the genome markers in their linear order; (d) SynBrowser; and (e) D-Genies [30], which works on the basis of alignment of genomes by minimap2 software package [31].

Fig. 2d illustrates similarities between *Xanthomonas oryzae* pv. *oryzae* PXO99A and *Xanthomonas oryzae* pv. *oryzae* MAFF 311018, 2 strains of *Xanthomonas oryzae* pv. *oryzae* (Xoo) pathogen, which causes the disease of bacterial blight of rice (*Oryza sativa* L.). It is the most serious bacterial disease of rice and can reduce yields by as much as 50% [25]. Note that to have a clearer picture, we have not plotted the shades connecting similar regions. This can be achieved using the “-l 6” option while calling the Smash++ visualizer. Figure S4 of the supplementary material provides the comparison of Smash++ with progressiveMauve and Salzberg et al. [25], which uses an alignment method to find genome rearrangements in Xoo. As can be seen, the result provided by Smash++ conforms to the one presented by Salzberg et al. [25], without performing an alignment.

### Comparison with Smash

**Figure 3:**
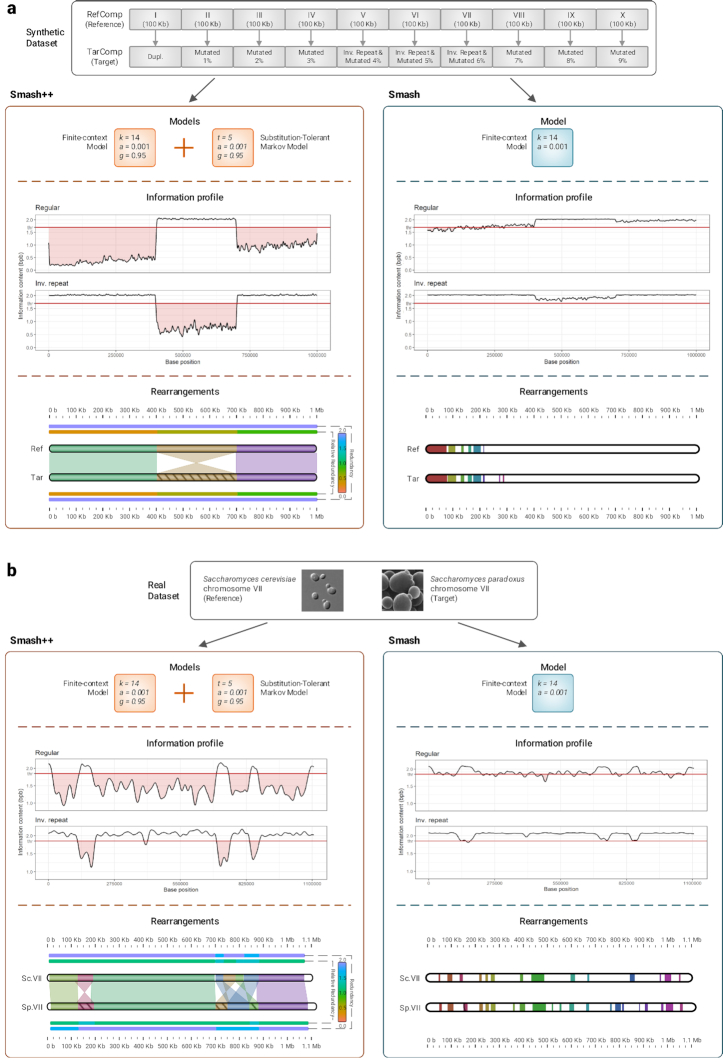
Comparison of Smash++ and Smash. (a) Performance on a synthetic dataset. Using the combination of an FCM and an STMM (in Smash++) produces more accurate results than using a single FCM (in Smash). (b) Performance on a real dataset, including *S. cerevisiae* chr. VII and *S. paradoxus* chr. VII. The rearrangement maps clearly show the improvement made over Smash, using an FCM along with an STMM.

To better understand the improvement we have made over the first version, Smash, we compare the 2 tools on a synthetic and a real dataset (see Fig. 3). In Table 1, the procedure for creating the synthetic data (RefComp and TarComp) is described. Fig. 3a compares running Smash and Smash++ on the synthetic dataset. For Smash, we used an FCM with *k*-mer size of 14, and for Smash++, we used a combination of an FCM with *k*-mer size of 14 and an STMM with number of substitutions of 5. As the information profiles show, Smash++ is better able to model the data because it uses less information (lower information content) to describe the target based on the reference; this is possible because of using a combination of the FCM and the STMM instead of using solely an FCM. We expect the output to have the following format: Parts I, II, III, and IV of the reference and the target are similar (including rearrangements); there are also inverted repeats between Parts V, VI, and VII of the sequences; and finally, there are rearrangements between Parts VIII, IX, and X of the sequences. When there is no space between consecutive regions, Smash++ joins them; therefore, we expect Smash++ to detect 3 similar regions: the one including Parts I, II, III, and IV; the one with Parts V, VI, and VII; and the one including Parts VIII, IX, and X. The rearrangement map shows that Smash++ fulfills our expectation. On the other side, Smash was not able to detect all rearrangements, showing that to model such a dataset, we need more than a single FCM.

The result of running Smash and Smash++ on a real dataset, *Saccharomyces cerevisiae* chromosome VII and *Saccharomyces paradoxus* chromosome VII, is demonstrated in Fig. 3b. *S. cerevisiae* is a species of yeast that plays a key role in winemaking, baking, and brewing. Its use as a eukaryotic model organism has provided insights into the molecular functioning of human cells [32]. *S. paradoxus* is the closest known species to *S. cerevisiae*, which has proved its importance in different fields of the life sciences, including evolution, ecology, and biotechnology [33]. For the experiment, we ran Smash using an FCM with *k*-mer size of 14, and Smash++ using an FCM with *k*-mer size of 14 combined with an STMM with number of substitutions of 5. As can be seen, using an FCM along with an STMM could drastically improve modeling the data, which led to finding rearrangements more accurately. The rearrangement map of Smash++ conforms to the previous study [32].

### Robustness against fragmented data

Inherited from Smash, Smash++ is capable of finding similarities between a fragmented reference and a target sequence. Figure S5 of the supplementary material shows the robustness of the proposed tool against fragmented data, for different randomly permutated block sizes. As can be seen, the same 3 target regions are detected even when the reference is fragmented to 100,000 blocks of 30 bp. This capability might be of interest in the case of non-assembled sequences or in the presence of assembly errors; note that this approach cannot be considered as an alternative to assembly.

### Benchmarking

**Figure 4:**
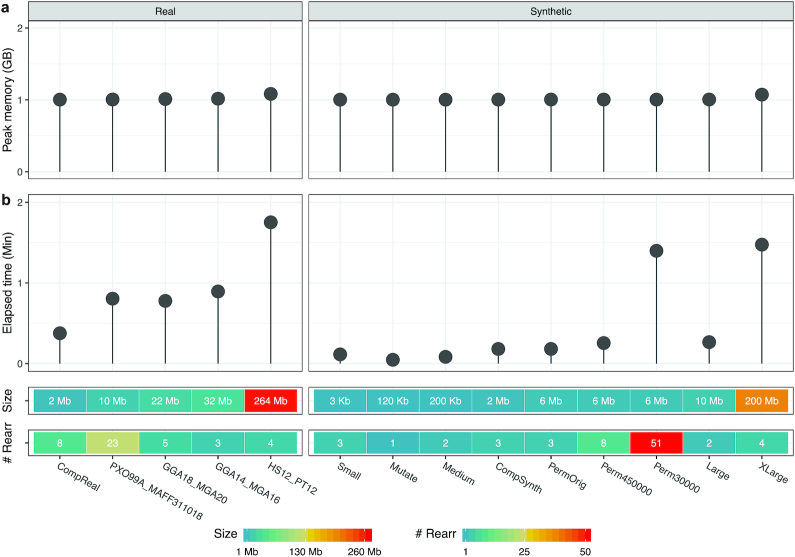
(a) The peak memory consumption, in gigabytes; and (b) the elapsed (wall clock) time usage, in minutes, of Smash++ obtained by running on all synthetic and real datasets described in Table 1.

Fig. 4 illustrates the performance of the proposed tool in terms of memory and time usage for all datasets (see supplementary Table S1 for more details). Size of the datasets and number of detected similar regions between each pair of sequences (“# Rearr”) are shown at the bottom of the figure. The pair dataset “Perm30” and the pair “Perm1000” are, as outliers, not shown. Fig. 4a shows the peak memory in gigabytes used by Smash++ on all synthetic and real datasets. As can be seen, it is ∼1 GB for all datasets. The maximum peak memory, ∼1.08 GB, was used when the proposed tool was run on human and chimpanzee chromosomes 12. It should be mentioned that the memory usage of Smash++ is related to the *k*-mer size that is used to model the data because different data structures are used for different *k*-mer sizes (see Methods). Sizes 13 and 14 were used to perform the experiments. The maximum memory usage of ∼1 GB enables Smash++ to run on any computer nowadays.

Fig. 4b demonstrates elapsed (wall clock) times, in minutes. The elapsed times rely on the file sizes along with the number of detected similar regions, meaning that the greater the number of regions and/or the greater the dataset size, the more time will be taken. Note that it is not a linear relation. As an example, the pair dataset “Large” and the pair “PXO99A_MAFF311018” have approximately the same total size of 10 Mb; but in the former case that 2 similarities is detected, Smash++ takes ∼16 seconds, and in the latter case with 23 similarities (∼12 times more than the former case), the proposed tool takes ∼48 seconds (3 times more) to run. As another example, carrying out Smash++ on the pair “Large” with 50 times larger size than the pair “Medium” leads to detection of the same number of rearrangements, i.e., 2, while it takes only ∼3 times more time. Regarding the pair “Perm30” with 11,565 similarities detected (Supplementary Table S1), we note that it has a massively fragmented reference sequence with 10,000 fragments of 30 bp; therefore it is by far the most time-consuming dataset. Note that the difference between the values of 10,000 (number of reference fragments) and 11,565 (number of similar regions) arises from the fact that a number of the reference chunks are similar to >1 target region and vice versa.

**Figure 5:**
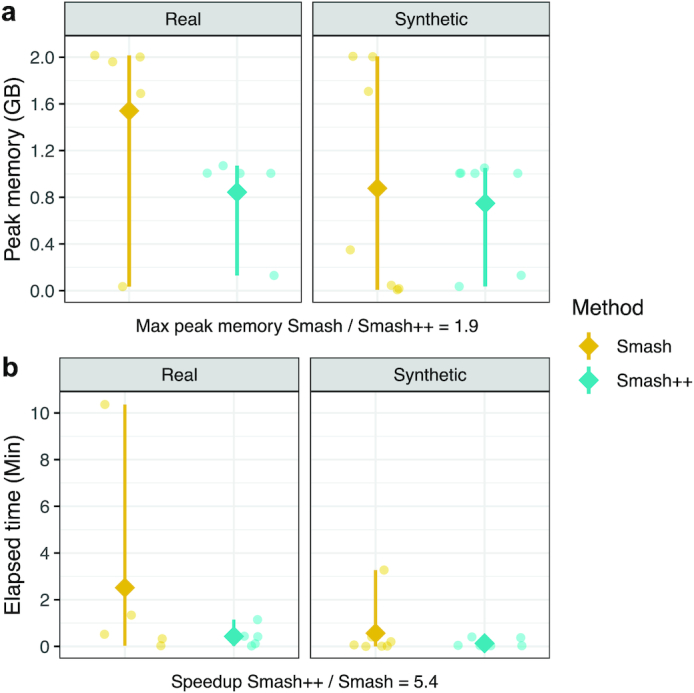
Comparison of Smash++ and Smash, in terms of (a) memory usage; and (b) time usage, running on real and synthetic data described in Table 1. To have a fair comparison, only 1 model (FCM) is used by Smash++, and also self-complexity is not computed. Diamonds indicate the mean, and bars, the ranges from minimum to maximum values.

A major advantage of Smash++ over Smash is using a combination of FCMs and STMMs to better model the data; however, to have an idea about how the performance of these 2 tools can be compared, we let Smash++ run with only 1 FCM on the dataset described in Table 1. We also did not compute self-complexity, similarly to Smash. Fig. 5 and supplementary Table S2 show the results. In Fig. 5a, the range of peak memory usages (from minimum to maximum usage) are compared, while running Smash and Smash++ on different real and synthetic datasets. The diamond symbol shows the mean value. The results show that the maximum peak memory usage by Smash++ is 1.9 times less than that by Smash. In Fig. 5b, the range of wall clock times is compared for these 2 tools. As mentioned in the figure, Smash++ runs 5.4 times faster than Smash on the tested datasets. It is worth mentioning that due to the absence of another tool that provides relative compression in addition to detecting rearrangements, we cannot have a fair quantitative camparison to other tools, in terms of time and memory usage; therefore, we have only included the results obtained by Smash++ and Smash.

## Conclusions

Finding genomic rearrangements is crucial becaue they play an important role in genetic disorders, cancer, and chromosomal evolution. We presented Smash++, an AF tool that accurately finds small- and large-scale genomic rearrangements between pairs of DNA sequences, by using a data compression approach. This memory-efficient tool was successfully tested on several synthetic and real data from bacteria, fungi, Aves, and Mammalia. The presented results show that the detected rearrangements were in accordance with previous studies, which used alignment-based methods or performed FISH analysis. Smash++ consumed a maximum of ∼1 GB of memory, among all experiments, which showed that it can be run on any computer, nowadays. The proposed tool has the potential to improve the accuracy of diagnostic and genetic counselling and also to guide future investigations into the development of personalized therapeutics.

## Methods

**Figure 6:**
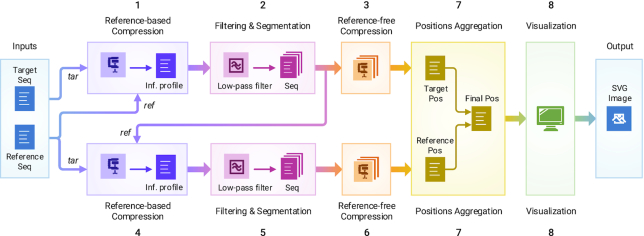
The schema of Smash++. The process of finding similar regions in reference and target sequences and computing the redundancy in each region includes 8 stages. Smash++ outputs a *.pos file that includes the positions of the similar regions, and can be then visualized, resulting in an SVG image.

The schema of the proposed method is illustrated in Fig. 6. Smash++ takes as inputs a reference and a target sequence and produces as output a position file, including local similarities of the 2 sequences, which can then be used by the Smash++ visualizer to produce an SVG image illustrating the similarities. This process has 8 major stages: (1) compression of the original target file, based on the model of the original reference file; (2) filtering the information profile, which is the output of Stage 1, and segmenting the target sequence; (3) reference-free compression of the segmented sequences obtained by the previous stage; (4) compression of the original reference file, based on the model of segmented sequences, which are obtained by Stage 2; (5) filtering the information profile and segmenting the reference sequence; (6) reference-free compression of the segmented sequences; (7) aggregating positions that are generated by stages 3 and 6; and (8) visualizing the positions. The following sections describe the process in detail.

### Data modeling

**Figure 7:**
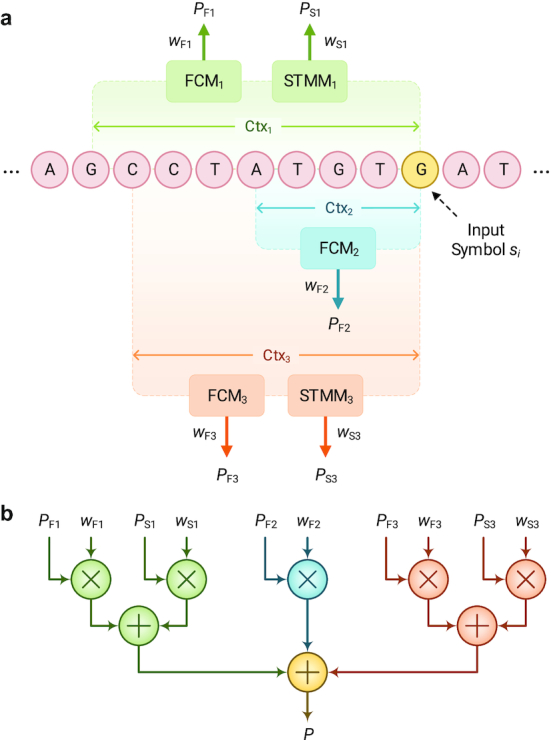
Data model used by Smash++. (a) Cooperation between finite-context models (FCMs) and substitution-tolerant Markov models (STMMs). Note that each STMM needs to be associated with an FCM. (b) Probability of an input symbol is estimated by using the probability and weight values that have been obtained from processing previous symbols.

We consider sequences over the nucleotide alphabet Θ = {A, C, G, T}; our goal is to measure the degree of local similarity between 2 such sequences. More specifically, we consider a reference sequence *S* = *s*_1_, …, *s_N_* over Θ, and we want to measure the local information content of a target sequence, given this reference sequence. To this end, we use a combination of finite-context models and substitution-tolerant Markov models to derive different probability measures for observing a nucleotide *x* in a sequence, given the context of the previous *k* nucleotides (Fig. 7a); these probabilities are then mixed (by multiplications and additions shown in Fig. 7b) to provide the final probability (*P*) of observing the nucleotide *x*. The following subsections describe the models we use in detail.

#### Finite-context model

We consider the probability of observing a certain nucleotide, given the previous *k* nucleotides, by using the relative frequency of this event in the reference sequence *S*. For *x* ∈ Θ and a *k*-mer *Q* ∈ Θ^*k*^, let *N*(*x*|*Q*) be the number of occurrences of *Q* in *S* that are followed by nucleotide *x*, and let *N*(*Q*) be the number of occurrences of *Q* in *S*. As in [23, 34, 35], we then define

(1) $$\begin{equation*}
P_{\text{FCM}} (x|Q) = \frac{N(x|Q) + \alpha }{N(Q) + 4 \cdot \alpha },
\end{equation*}$$

where “4” is the size of alphabet Θ and α is a pseudo-count parameter. For α = 1, Eq. 1 turns into the Laplace estimator. Note that an FCM has the Markov property, in which the conditional probability distribution of observing a nucleotide depends only on the state of the preceding *k*-mer.

#### Substitution-tolerant Markov model

Given the reference sequence *S*, we use the aforementioned probability distribution *P*_FCM_ to define a sequence $S^{\prime } = s^{\prime }_{-k}, s^{\prime }_{-k+1}, \dots , s^{\prime }_N$ recursively by

(2) $$\begin{equation*}
s^{\prime }_i = \left\lbrace \begin{array}{ll}\text{A} & \text{if } i \lt 1 \\
\displaystyle \mathop {\mathrm{arg\, max}}_{x\in \Theta } P_{\text{FCM}}(x|s^{\prime }_{i-k}, \dots , s^{\prime }_{i-1}) & \text{if } i \ge 1. \\
\end{array} \right.
\end{equation*}$$

For *x* ∈ Θ and a *k*-mer *Q* ∈ Θ^*k*^, we then define *N*′(*x*|*Q*) as the number of occurrences of *Q* followed by *x* and *N*′(*Q*) as the number of occurrences of *Q*, respectively, in the sequence *S*′. Finally, we define

(3) $$\begin{equation*}
P_{\text{STMM}}(x|Q) = \frac{N^{\prime }(x|Q) + \alpha }{N^{\prime }(Q) + 4 \cdot \alpha }.
\end{equation*}$$

STMMs, which are probabilistic-algorithmic models [23, 36], can be used along with FCMs to modify the behavior of Smash++ when confronted with nucleotide substitutions in genomic sequences. These models can be disabled, to reduce the number of mathematical calculations, and consequently, increase the performance of the proposed method. Such an operation is automatically performed using an array of size *k* (the context size), named "history," which preserves the past *k* hits/misses. Observing a symbol in the sequence, the memory is checked for the symbol with the highest number of occurrences. If they are equal, a hit is saved in the history array; otherwise, a miss is inserted into the array. Before getting to store a hit/miss in the array, it is checked for the number of misses and in the case they are more than a predefined threshold *t*, the STMM will be disabled and also the history array will be reset. This process is performed for each nucleotide in the sequence.

The following example shows the distinction between an FCM and an STMM. Assume that the current context at a certain position is AGACGTAC, and the number of occurrences of symbols saved in memory is 10, 6, 15, and 8 for A, C, G, and T, respectively; also, the next symbol to appear in the sequence is T. An FCM considers the next context as GACGTAC**T**, while an STMM considers it as GACGTAC**G** because the nucleotide G is the most probable symbol, based on the number of occurrences stored in memory.

#### Cooperation of FCMs and STMMs

When FCMs and STMMs are in cooperation, the probability of observing a nucleotide *x* in a sequence *S* can be estimated as

(4) $$\begin{equation*}
P(x) = \sum \nolimits_{i=1}^m P_{\text{FCM}_i}(x|Q)\,\,w_i + \sum \nolimits_{j=1}^n P_{\text{STMM}_j}(x|Q)\,\,w_j, \quad \forall x\in S,
\end{equation*}$$

in which *m* and *n* denote the number of FCMs and STMMs, respectively, and *w_i_* and *w_j_* are weights assigned to each FCM and STMM, respectively, based on its performance. We have

(5) $$\begin{equation*}
\begin{array}{ll}w_{i_p} \propto (w_{i_{p-1}})^{\gamma _i} P_{\text{FCM}}(x|Q_{p-1}), & 1\le i\le m, \\
[1mm] w_{j_p} \propto (w_{j_{p-1}})^{\gamma _j} P_{\text{STMM}}(x|Q_{p-1}), & 1\le j\le n, \end{array}
\end{equation*}$$

where *p* denotes a certain position, and γ_*i*_ and γ_*j*_ ∈ [0, 1) are forgetting factors predefined for each model. Also,

(6) $$\begin{equation*}
\sum \nolimits_{i=1}^m w_i + \sum \nolimits_{j=1}^n w_j = 1.
\end{equation*}$$

By experimenting with different forgetting factors and context-order sizes for models, we have found that the factors are directly related to the context sizes and reciprocally related to the complexity (see Fig. S6 of the supplementary material).

### Storing models in memory

The FCMs and STMMs include, in fact, count values that need to be saved in memory. For this purpose, 4 different data structures have been used considering the context-order size *k*, as follows:

- table of 64-bit counters, for 1 ≤ *k* ≤ 11,
- table of 32-bit counters, for *k* = 12, 13,
- table of 8-bit approximate counters, for *k* = 14, and
- Count-Min-Log Sketch (CMLS) of 4-bit counters, for *k* ≥ 15.

**Figure 8:**
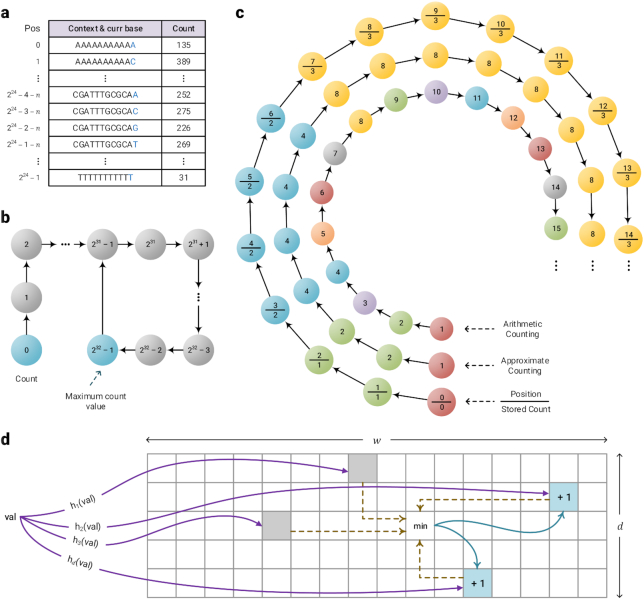
The data structures used by Smash++ to store the models in memory. (a) Table of 64-bit counters that uses up to 128 MB of memory, (b) table of 32 bit counters that consumes at most 960 MB of memory, (c) table of 8 bit approximate counters with memory usage of up to 1 GB, and (d) Count-Min-Log sketch of 4-bit counters, which consumes up to $\frac{1}{2} w\times d$ B of memory; e.g., if *w* = 2^30^ and *d* = 4, it uses 2 GB of memory.

The table of 64-bit counters (Fig. 8a) simply saves the number of events for each context. The table of 32-bit counters saves in each position the number of times that the associated context is observed. When a counter reaches the maximum value 2^32^ − 1 = 4,294,967,295, all the counts will be renormalized by dividing by 2, as shown in Fig. 8b.

**Figure 9:**
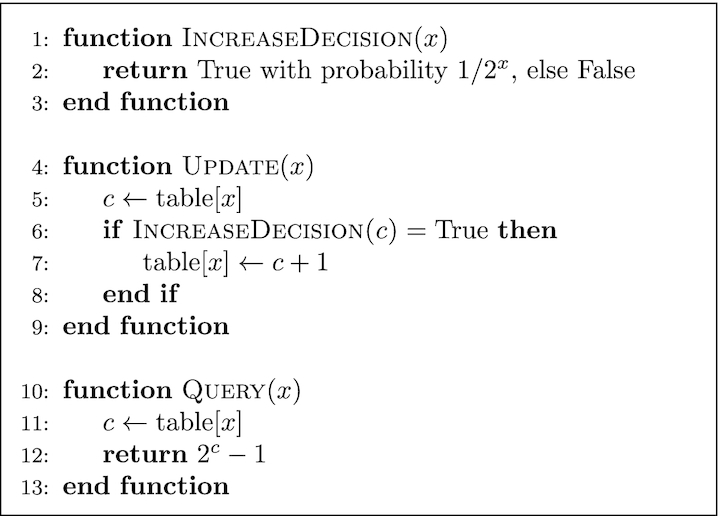
Approximate counting update and query.

Approximate counting is a method that use probabilistic techniques to count large number of events while using a small amount of memory [37]. Fig. 9 shows the algorithm for 2 major functions associated with this method, Update and Query. To update the counter, a pseudo-random number generator is used the number of times of the counter’s current value to simulate flipping a coin. If it comes up 0/Heads each time or 1/Tails each time, the counter will be incremented. Fig. 8c shows the difference between arithmetic and approximate counting, and also the values that are actually stored in memory. Note that because an approximate counter represents the actual count by an order-of-magnitude estimate, one only needs to save the exponent. For example, if the actual count is 8, we store in memory log_2_8 = 3.

**Figure 10:**
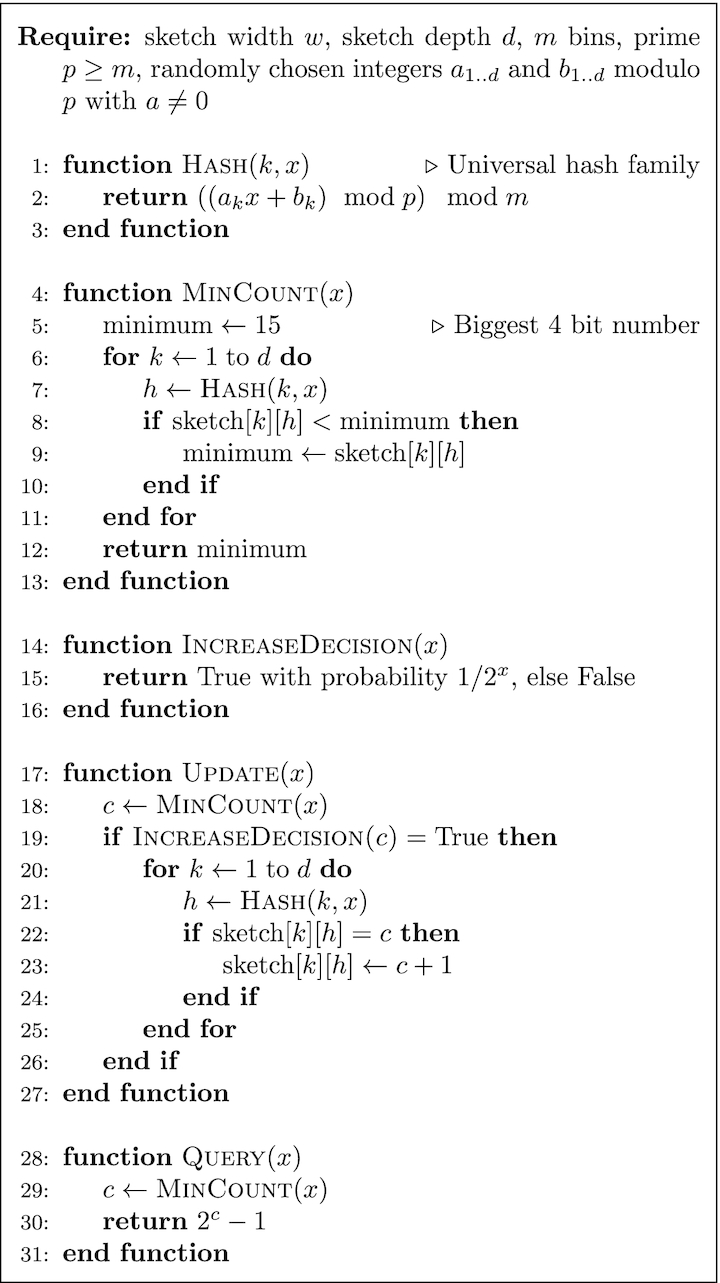
Count-Min-Log Sketch update and query.

CMLS is a probabilistic data structure to save the frequency of events in a table by means of a family of independent hash functions [38]. The algorithm for updating and querying the counter is shown in Fig. 10. To update the counter, its current value is hashed with *d* independent hash functions. Then, a coin is flipped the number of times of the counter’s current value, using a pseudo-random number generator. If it comes up 0/Heads each time or 1/Tails each time, the minimum hashed values (out of *d* values) will be updated, as shown in Fig. 8d.

CMLS requires a family of pairwise independent hash functions *H* = {*h*: *U* → [*m*]}, in which each function *h* maps some universe *U* to *m* bins. In this family of functions, the probability that all *x, y* ∈ *U*,  *x* ≠ *y* will hash to any pair of hashed values *z*_1_, *z*_2_ is as if they were perfectly random, i.e., *P*_*h* ∈ *H*_[*h*(*x*) = *z*_1_∧*h*(*y*) = *z*_2_] = 1/*m*^2^. A hash function in this family can be obtained by

(7) $$\begin{equation*}
h_{a,b}(x) = \left(\left(ax+b\right)~\bmod p\right)~\bmod m,
\end{equation*}$$

where *p* ≥ *m* is a prime number and *a* and *b* are randomly chosen integers modulo *p* with *a* ≠ 0. Note that if the number of bins is a power of 2, *m* = 2^*M*^, the multiply-add-shift scheme [39] can be used to avoid modular arithmetic. A hash function in this scheme can be obtained by:

(8) $$\begin{equation*}
h_{a,b}(x) = \left(\left(ax+b\right)~\bmod 2^w\right)~\mathrm{div}~2^{w-M},
\end{equation*}$$

in which *w* is the number of bits in a machine word, e.g., 64; *a* is a random positive integer <2^*w*^; and *b* is a random non-negative integer <2^*w* − *M*^. Such a hash function can be implemented in the C++ language by

$$\begin{equation*}
h_{a,b}(x) = (\mathrm{uint64\_t})~(a\star x+b)~\gg ~(w-M).
\end{equation*}$$

### Finding similar regions

To find similar regions in reference and target sequences, a quantity is required for measuring the similarity. We use “per symbol information content,” in bpb (bits per base), which can be calculated as

(9) $$\begin{equation*}
I(x) = -\log _2 P(x), \quad \forall x\in S,
\end{equation*}$$

where *P*(*x*) denotes the probability of observing a nucleotide *x* in the sequence *S*, obtained by Equation 4.

The information content is the amount of information required to represent a symbol in the target sequence, based on the model of the reference sequence. The less the value of this measure is for 2 regions, the more information is shared between them, and, therefore, the more similar are the 2 regions. Note that a version of this measure has been introduced by Pratas et al. [21], who used a single FCM to calculate the probabilities. In this article, however, we exploit a cooperation between multiple FCMs and STMMs for highly accurate calculation of such probabilities.

**Figure 11:**
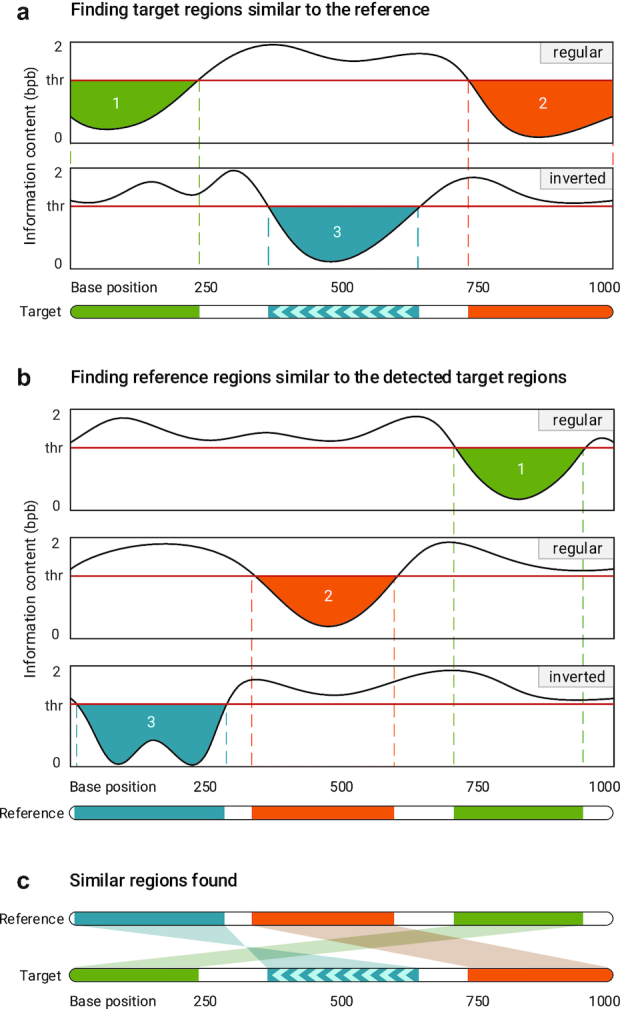
Finding similar regions in reference and target sequences. Smash++ first finds the regions in the target that are similar to the reference and then finds the regions in the reference that are similar to the detected target regions. This procedure is performance for both regular and inverted homologies.

The procedure of finding similar regions in a reference and a target sequence, illustrated in Fig. 11, is as follows: after creating the model of the reference, the target is compressed based on that model and the information content is calculated for each symbol in the target. Then, the content of the whole target sequence is smoothed by a Hann window [40], which is a discrete window function given by $w[n]=0.5-0.5\,\,\cos \left(2\pi n/N\right)$, where 0 ≤ *n* ≤ *N* and length of the window is *N* + 1. Next, the smoothened information content is segmented considering a predefined threshold, meaning that the regions with content greater than the threshold are filtered out. This is carried out for both regular and inverted repeat homologies, and, at the end, the result would be the regions in the target sequence that are similar to the reference sequence (Fig. 11a). The described phase repeats for all of the target regions found, in such a way that after creating the model for each region, the whole reference sequence is compressed to find those regions in the reference that are similar to each of the target regions (Fig. 11b). The final result would have the form of Fig. 11c.

### Computing complexity

After finding the similar regions in reference and target sequences, we evaluate redundancy in each region, knowing that it is inversely related to Kolmogorov complexity, i.e., the more complex a sequence is, the less redundant it will be [41]. The Kolmogorov complexity, *K*, of a binary string *s*, of finite length, is the length of a shortest binary program *p* that computes *s* in a universal Turing machine and halts. In other words, *K*(*s*) = |*p*| is the minimum number of bits required to computationally retrieve the string *s* [42, 43].

The Kolmogorov complexity is not computable; hence, an alternative is required to compute it approximately. It has been shown in the literature that a compression algorithm can be used for this purpose [44–46]. In this article, we use a reference-free compressor to approximate the complexity and, consequently, the redundancy of the found similar regions in the reference and the target sequences. This compressor works on the basis of cooperation of FCMs and STMMs, which has been previously described in detail. Note that the difference between a reference-based and reference-free version of such a compressor is that, in the former mode, a model is first created for the reference sequence and then the target sequence is compressed on the basis of that model, while in the latter mode, the model is progressively created at the time of compressing the target sequence.

## Availability of Source Code and Requirements

Project name: Smash++

Project home page: https://github.com/smortezah/smashpp

Operating system(s): Linux, macOS, Windows

Programming language: C++, Python

Other requirements: C++ 14, Python 3

License: GNU GPLv3

RRID: SCR_018307

## Availability of Supporting Data and Materials

The datasets supporting the results of this article are available in the Smash++ Github repository, https://github.com/smortezah/smashpp/tree/master/experiment/dataset. Snapshots of our code and other supporting data are available in the *GigaScience* repository, GigaDB [47].

## Additional Files

**Supplementary Figure S1**. Comparison of Smash++ and other methods on *G. gallus* chromosome 18 and *M. gallopavo* chromosome 18.

**Supplementary Figure S2**. Different methods running on *G. gallus* chromosome 14 and *M. gallopavo* chromosome 16.

**Supplementary Figure S3**. Comparing with other methods on *H. sapiens* chromosome 12 and *P. troglodytes* chromosome 12.

**Supplementary Figure S4**. Result of running different methods on *X. oryzae* pv. *oryzae* PXO99A and *X. oryzae* pv. *oryzae* MAFF 311018.

**Supplementary Figure S5**. Similarity of a target sequence to a fragmented reference sequence, which is randomly permutated by different block sizes.

**Supplementary Figure S6**. Relation between context-order sizes, forgetting factors, and complexity (information content).

**Supplementary Table S1**. Performance of Smash++, in terms of memory and time usage, running on different synthetic and real datasets.

**Supplementary Table S2**. Comparison of the performance of Smash++ and Smash, running on different synthetic and real datasets.

**Supplementary Note S1**. Software manual for Smash++.

giaa048_GIGA-D-20-00010_Original_Submission

giaa048_GIGA-D-20-00010_Revision_1

giaa048_GIGA-D-20-00010_Revision_2

giaa048_Response_to_Reviewer_Comments_Original_Submission

giaa048_Response_to_Reviewer_Comments_Revision_1

giaa048_Reviewer_1_Report_Original_Submission Alexander Peltzer -- 2/12/2020 Reviewed

giaa048_Reviewer_2_Report_Original_Submission Johannes Zuegg -- 2/23/2020 Reviewed

giaa048_Supplemental_File

## Abbreviations

AF: alignment-free; bp: base pairs; CMLS: Count-Min-Log Sketch; CPU: central processing unit; FCM: finite-context model; FISH: fluorescence *in situ* hybridization; GGA:   *Gallus gallus*; HS: *Homo sapiens*; HSV: hue, saturation, value; HTS: high-throughput sequencing; Mb: megabase pairs; MGA: *Meleagris gallopavo*; MYA: million years ago; NCBI: National Center for Biotechnology Information; PMP22: peripheral myelin protein 22; PT: *Pan troglodytes*; RAM: random access memory; Sc: *Saccharomyces cerevisiae*; Sp: *Saccharomyces paradoxus*; STMM: substitution-tolerant Markov model; SVG: Scalable Vector Graphics; UCSC: University of California, Santa Cruz.

## Competing Interests

The authors declare that they have no competing interests.

## Funding

M.H. was supported by PhD MAP-i grant (PD/BD/113969/2015) from Foundation for Science and Technology (FCT) in Portugal. D.P. was funded by Scientific Employment Stimulus Program (CI-CTTI-94-ARH/2019) from FCT. M.H., D.P., and A.J.P. were supported by Operational Program of Competitiveness and Internationalization (COMPETE) (UID/CEC/00127/2019 and UIDB/00127/2020) from FCT.

## Authors' Contributions

M.H. developed the software and wrote the manuscript. D.P. and A.J.P. contributed to and tested the software. D.P., B.M., and A.J.P. provided guidance. All authors review the final manuscript and provide critical comments.

## Acknowledgements

We thank everyone who has contributed to the development of Smash++, through testing and feedback.
