# Supplementary material for: Smash++: an alignment-free and memory-efficient tool to find genomic rearrangements
Source: Gigascience. 2020 May 20;9(5):giaa048. doi: 10.1093/gigascience/giaa048 (PMC7238676; doi:10.1093/gigascience/giaa048)
Supplement: giaa048_Supplemental_File [file giaa048_supplemental_file.pdf]

Supplementary Material for

Smash++: an alignment-free and memory-efficient tool to find genomic rearrangements

Morteza Hosseini, Diogo Pratas, Burkhard Morgenstern, Armando J. Pinho

seyedmorteza@ua.pt pratas@ua.pt bmorgen@gwdg.de ap@ua.pt

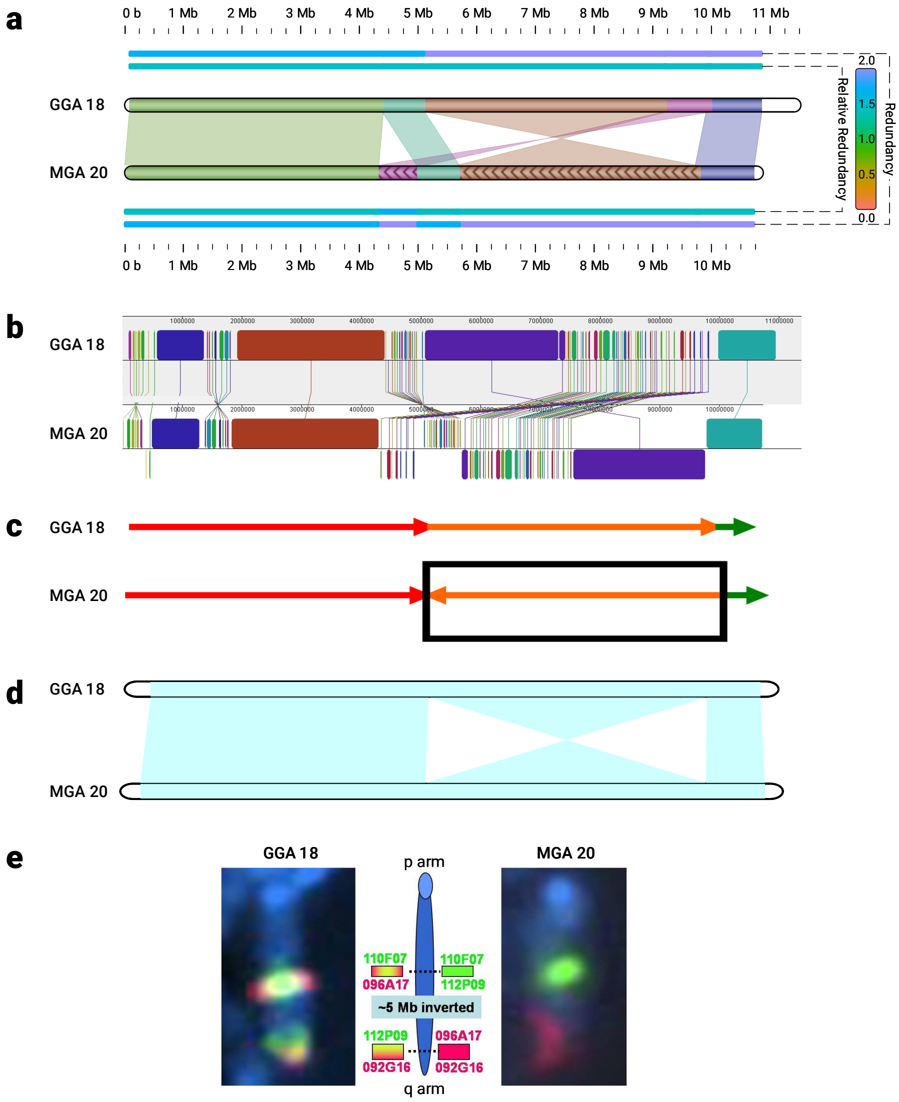

Figure S1. Pair-wise comparison of *G. gallus* chromosome 18 and *M. gallopavo* chromosome 20. (a) Smash++, with $k = 14$ and 5 used by an FCM and an STMM, respectively. The blocks smaller than 500 Kb are discarded; (b) progressiveMauve [1], with LCB (locally collinear block) weight of 18692. Reverse complements are shown in lower level; (c) adopted from [2], which is confirmed by fluorescence *in situ hybridization* (FISH) analysis. The box shows a local rearrangement; (d) SynBrowser [3], with the resolution of 150 Kb (minimum size of a reference block); (e) adopted from [4], which confirms an inversion rearrangement of size ∼5 Mb by FISH analysis.

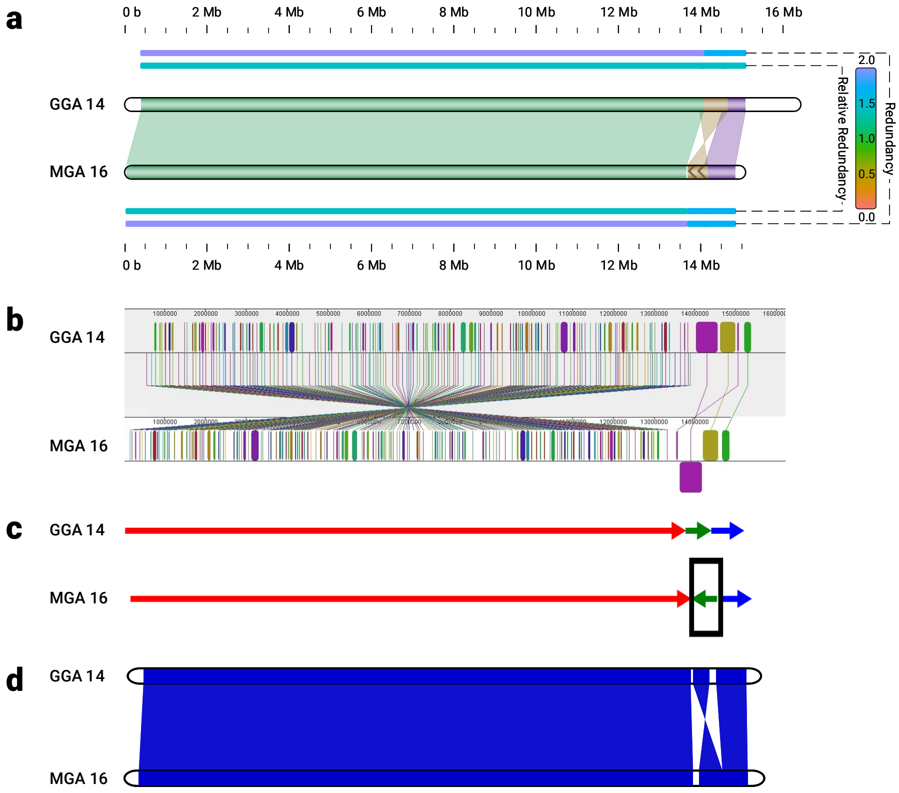

Figure S2. *G. gallus* chromosome 14 compared to *M. gallopavo* chromosome 16. (a) Smash++, employing an FCM and an STMM with $k = 14$ and 5, respectively. The blocks smaller than 400 Kb are discarded; (b) progressiveMauve, with LCB weight of 27424; (c) adopted from [2]. The box shows an inversion rearrangement; (d) SynBrowser, with the resolution of 150 Kb.

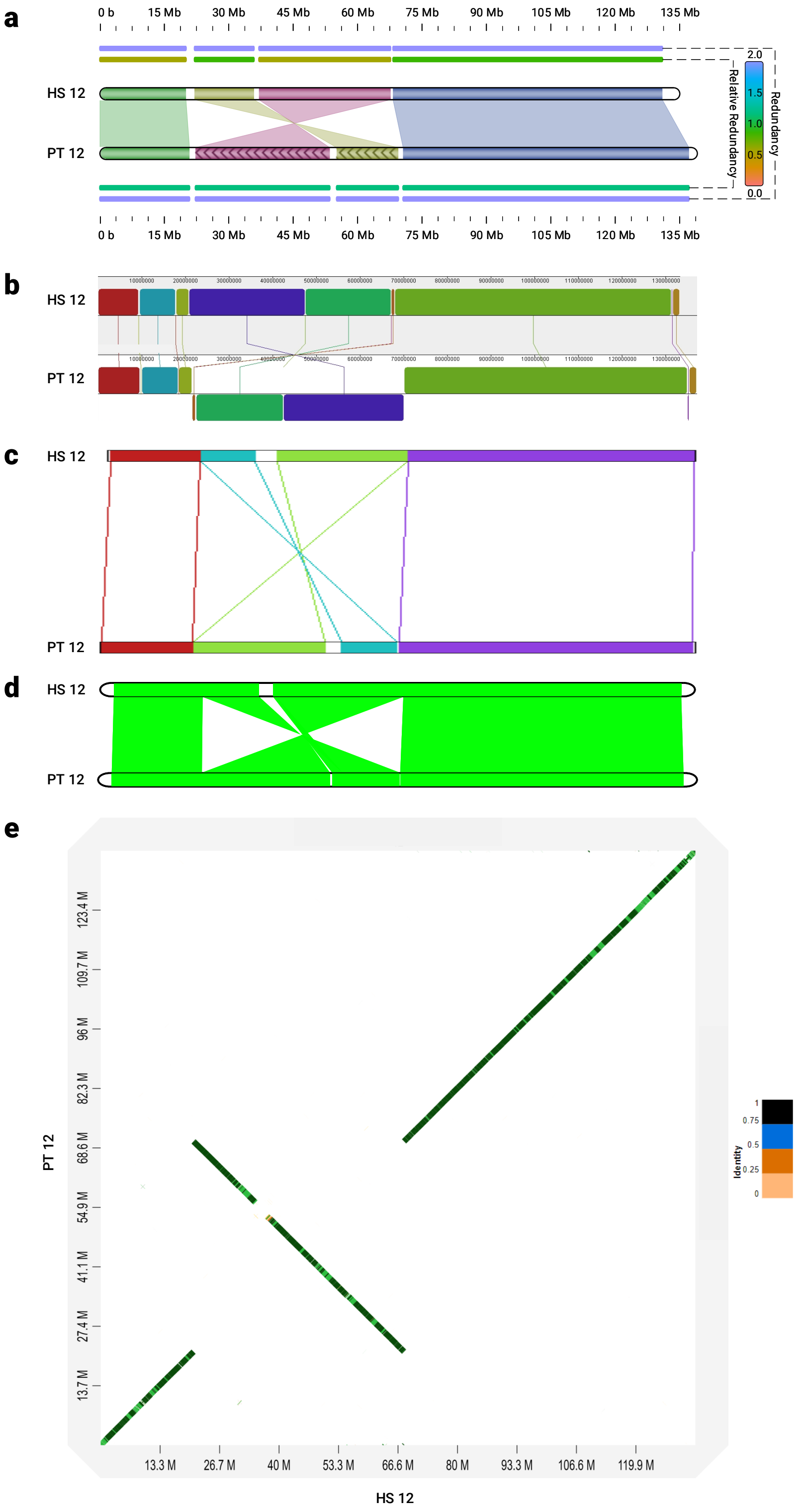

Figure S3. Comparison of *H. sapiens* chromosome 12 and *P. troglodytes* chromosome 12. (a) Smash++, with $k = 14$ used by an FCM. The blocks smaller than 100 Kb are discarded; (b) progressiveMauve, with LCB weight of 55186; (c) Cinteny [5], with minimum length of synteny block = 1 Kb, maximum gap between adjacent markers = 5 Mb and minimum number of markers = 1; (d) SynBrowser, with the resolution of 150 Kb; (e) D-GENIES [6], in “strong precision” mode.

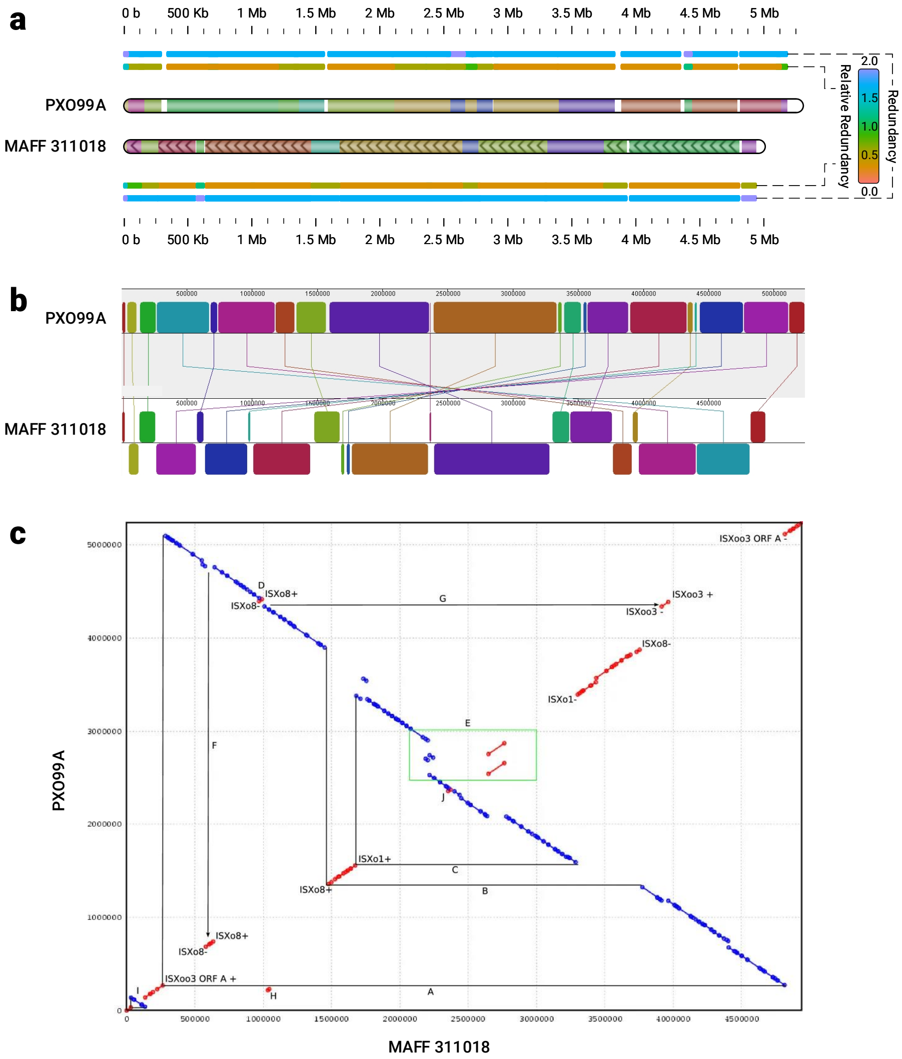

Figure S4. Pair-wise comparison of PXO99A and MAFF 311018. (a) Smash++, with k=13 used by an FCM. The blocks smaller than 10 Kb are discarded. In order to make the figure clearer, the shaded paths for connecting corresponding regions are not drawn; (b) progressiveMauve, with LCB weight of 3926; (c) adopted from [7], which employs an alignment-based method to obtain this dot plot. The blue and red colors shows regions of PXO99A that align to the same or opposite strand of MAFF 311018, respectively.

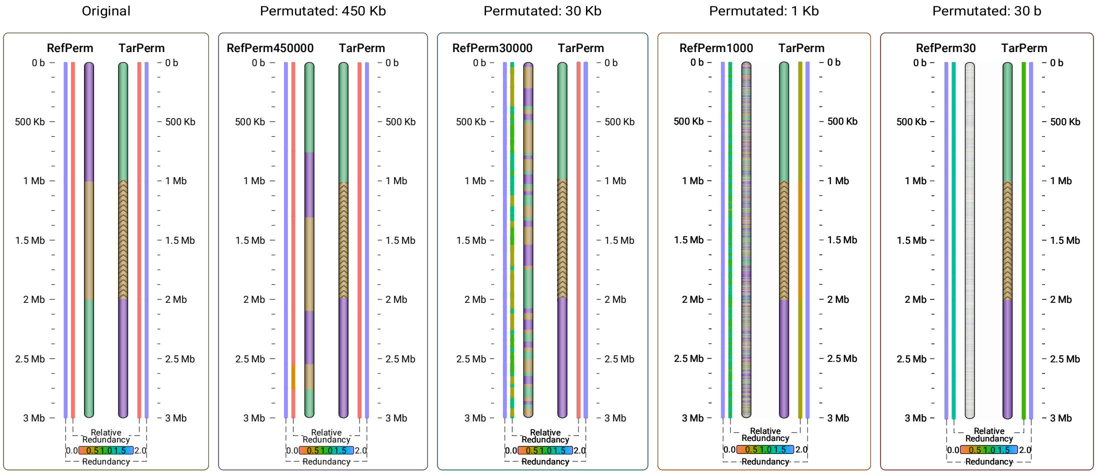

Figure S5. Similarity of a target sequence to a reference sequence, when the reference is permutated by blocks of different sizes of 450 Kb, 30 Kb, 1 Kb and 30 b. To run Smash++, an FCM with k-mer size of 14 and the threshold of 1.5 was used. It shows the proposed method is resistant to fragmentation of sequence; for example, in the case of 30 b, although the reference is highly fragmented, Smash++ could detect the same three similar regions in the target as the original case.

Table S1. Performance of Smash++, in terms of memory and time usage, running on all synthetic and real datasets, described in Table 1.

Dataset (Reference + Target)	Size (b)	# Rearr	Memory (KB)	Elapsed time (s)	User time (s)	System time (s)
Synthetic						
Small	3000	3	1,053,528	6.95	1.76	5.22
Medium	200,000	2	1,053,328	5.11	1.45	3.68
Large	10,000,000	2	1,055,860	16.06	12.20	3.73
XLarge	200,000,000	4	1,126,892	88.61	78.67	7.95
Mutate	120,000	1	1,053,308	2.93	0.87	2.06
PermOrig	6,000,000	3	1,055,304	10.98	4.43	6.50
Perm450000	6,000,000	8	1,055,128	15.37	5.74	9.55
Perm30000	6,000,000	51	1,054,900	84.02	24.58	59.14
Perm1000	6,000,000	702	1,054,660	652.58	176.82	474.16
Perm30	6,000,000	11,565	1,057,804	24,958.90	16,542.13	7629.04
CompSynth	2,000,000	3	1,054,236	10.98	5.58	5.26
Real						
GGA14_MGA16	31,542,564	3	1,067,380	53.74	44.80	8.57
GGA18_MGA20	22,419,393	5	1,062,700	46.75	37.77	8.73
HS12_PT12	264,271,225	4	1,137,216	105.21	95.15	7.36
PXO99A_MAFF311018	10,324,186	23	1,056,048	48.39	16.24	31.86
CompReal	2,228,294	8	1,054,532	22.66	9.21	13.35

Table S2. The memory and time usage of Smash++ and Smash, running on different synthetic and real datasets, described in Table 1. To have a fair comparison, Smash++ uses only one model (FCM), as Smash does. Note that the pair datasets "Perm450000", "Perm30000", "Perm1000" and "Perm30" were excluded from this experiment, since they were basically used to show the potential of Smash++ to handle fragmented data.

Dataset	Size (b)	Memory (KB)		Elapsed time (s)	
(Reference + Target)		Smash	Smash++	Smash	Smash++
Synthetic					
Small	3000	8440	1,053,416	0.03	2.14
Medium	200,000	366,624	1,053,560	0.95	1.77
Large	10,000,000	2,104,976	1,055,852	12.15	2.75
XLarge	200,000,000	2,101,496	1,102,036	196.29	24.36
Mutate	120,000	18,268	38,136	0.61	1.78
PermOrig	6,000,000	48,252	137,784	3.76	0.97
CompSynth	2,000,000	1,789,796	1,053,960	24.33	22.39
Real					
GGA14_MGA16	31,542,564	2,113,716	1,053,672	19.74	7.01
GGA18_MGA20	22,419,393	2,055,804	1,054,124	31.20	25.06
HS12_PT12	264,271,225	2,098,868	1,122,652	621.70	68.87
PXO99A_MAFF311018	10,324,186	1,771,048	1,055,612	80.38	26.23
CompReal	2,228,294	36,992	136,588	1.79	1.36

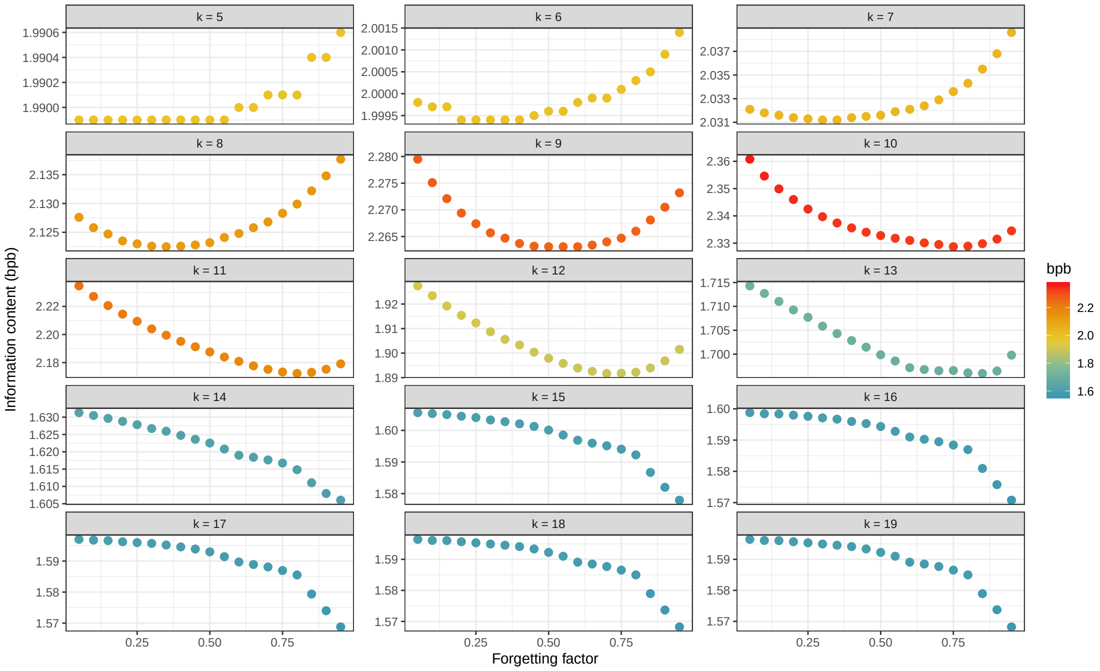

Figure S6. The relation between context-order sizes (k), forgetting factors and complexity (information content). It is shown that higher factors should be assigned to models that have higher context-order sizes (less complexity) and vice versa. For the experiment, we compressed 10 synthetic sequences with the sizes of 500 Kb to 20 Mb, with different redundancies, and calculated the average information content.

Note S1 Software manual

Smash++ is implemented in the C++ language and is publicly available at https://github.com/smortezah/smashpp, under GNU GPL v3 license. This tool is able to find and visualize rearrangements in a pair of DNA sequences. It is recommended to use as input bare sequences, i.e., without header or quality scores, although, FASTA and FASTQ formats are also supported. In the following sections, we describe installing and running the Smash++ tool.

S1.1 Install

To install Smash++ on various operating systems, follow the instructions below. Note that the precompiled executables are available for 64 bit operating systems in the "experiment/bin/" directory.

Conda

```
conda install -c bioconda smashpp
```

Linux

- Install "git" and "cmake":

```
sudo apt update
sudo apt install git cmake
```

- Clone Smash++ and install it:

```
git clone https://github.com/smortezah/smashpp.git
cd smashpp
./install.sh
```

macOS

- Install "Homebrew", "git" and "cmake":

```
/usr/bin/ruby -e "$(curl -fsSL https://raw.githubusercontent.com/Homebrew/
    install/master/install)"
brew install git cmake
```

- Clone Smash++ and install it:

```
git clone https://github.com/smortezah/smashpp.git
cd smashpp
./install.sh
```

Windows

After installing "wsl" (Windows Subsystem for Linux), the commands to install Smash++ would be the same as for Linux/macOS:

```
git clone https://github.com/smortezah/smashpp.git
cd smashpp
./install.sh
```

S1.2 Run

Various options are provided with the interface of the proposed tool, which are described in Table S3. The commands for running Smash++ have the form of the following:

```
./smashpp [OPTIONS] -r <REF-FILE> -t <TAR-FILE>
```

Table S3. Options provided by Smash++ interface.

Flag	Input	Description
Required		
`-r`	Seq/FASTA/FASTQ	Reference file.
`-t`	Seq/FASTA/FASTQ	Target file.
		Spaces in the names are supported. It is recommended to choose short names.
Optional		
`-l`	Integer: $[0, 6]$ Default: 3	Level of compression. The lower the level, the faster the Smash++ and the higher the level, the more accurate the Smash++.
`-m`	Integer: $[1, 2^{32} - 1]$ Default: 50	Minimum segment size. Only the regions that have greater sizes than this value would be considered for compression. On the one hand, if it is set to a very small value, e.g., 5, Smash++ might detect many tiny regions. Choosing a larger value leads to joining of the potential tiny regions. On the other hand, if it is set to a huge value, e.g., 1,000,000,000, many regions (probably all of them) will be discarded. Therefore, a value should be chosen in between by trial and error, with regard to the sequence size. Usually, higher values are more appropriate for larger sequences. To have a better understanding of the values that could be chosen, see the script "xp.py" at `https://github.com/smortezah/smashpp/blob/master/experiment/xp.py`.
`-e`	Float: $[0.0, 100.0]$ Default: 2.0	Entropy of 'N' bases. In implementation of the reference-based compression, we replace 'N's in references and targets with 'A's and 'T's, respectively. On reference-free compression, we replace them with 'A's, in both references and targets. If a user tends to replace 'N' bases in a sequence with a normal distribution of 'A', 'C', 'G' and 'T's, he/she can employ the GOOSE toolkit `https://github.com/pratas/goose`.
`-n`	Integer: $[1, 255]$ Default: 4	Number of threads. Creating multiple finite-context models and substitution-tolerant Markov models can be done in a multi-threaded fashion.
`-f`	Integer: $[1, 2^{32} - 1]$ Default: 256	Filter size. In the process of finding similar regions in the reference and the target sequences, the information content obtained by compression needs to be filtered. This value is directly dependant on the size of sequences. If it is set to a very small value, there would be many spikes on the information profile, which makes Smash++ detect many tiny regions. To have a better understanding of the values that could be chosen, see `https://github.com/smortezah/smashpp/blob/master/experiment/xp.py`.
`-ft`	Integer or String: {0/rectangular, 1/hamming, 2/hann, 3/blackman, 4/triangular, 5/welch, 6/sine, 7/nuttall} Default: hann	Filter type (windowing function). Besides Hann window that is used by default to smooth the information content (profile), we have implemented several other windowing functions: Blackman [8], Hamming [9], Nuttall [10], rectangular [11], sine [12], triangular [13] and Welch [14] windows. These functions along with Hann function are given by Equation S1 and are plotted in Fig. S7. Note that the rectangular function provides the fastest and the least accurate results.
`-fs`	Char or String: {S/small, M/medium, L/large}	Filter scale. It automatically chooses filter size. If a user does not use this flag, the "-f" flag will handle the filtering size.
`-d`	Integer: $[1, 2^{64} - 1]$	Sampling steps. Instead of considering the whole information content, we can make samples of it. The default value is $\lceil \min(\lvert\text{ref}\rvert, \lvert\text{tar}\rvert) / 5000 \rceil$. Therefore, if this flag is not entered by user, a maximum number of 5000 values of the information content will be considered.
`-th`	Float: $[0.0, 20.0]$ Default: 1.5	Threshold for entropy. This option can be used for segmenting the filtered information content. The more similar the two sequences are, the less the entropy is and therefore, a lower threshold can be set. Note that the threshold is not dependant on the sequence sizes.
`-rb`	Integer: $[-2^{15}, 2^{15}-1]$	Reference beginning guard.
`-re`	Integer: $[-2^{15}, 2^{15}-1]$	Reference ending guard.
`-tb`	Integer: $[-2^{15}, 2^{15}-1]$	Target beginning guard.
`-te`	Integer: $[-2^{15}, 2^{15}-1]$	Target ending guard.
	Default: 0	Smash++ is capable of finding even very small similar regions in two sequences. However, we have found experimentally that when it is running in a very sensitive

		mode, there might be some cases in which the size of similar regions in the reference and the target are not balanced. These cases can be handled by "-rb", "-re", "-tb" and "-te" options, by resizing the beginning and ending guards of reference and target regions, respectively. For example, if "-tb 10" is used, the first 10 bases in each target region will be discarded, which results in smaller regions. Note that when the guard sizes of target regions are increased, the models built from these regions would be slightly different than the original models; consequently, the sizes of reference regions that are detected as being similar to the ones from the target would be altered. Therefore, changing the guard sizes of target regions will affect the sizes of reference regions. The same thing happens for the sizes of target regions, when the guard sizes of reference regions are changed.
`-ar`	N/A<br>Default: not applied	Consider asymmetric regions. It makes Smash++ not to consider a balance between the sizes of similar reference and target regions. Hence, it allows a target region not to follow the following equation:<br>$0.5 * \text{size(ref region)} < \text{size(tar region)} < 1.5 * \text{size(ref region)}$.
`-nr`	N/A<br>Default: not applied	Do not compute self complexity. It makes the tool not to perform the reference-free compression (self-complexity computation).
`-sb`	N/A<br>Default: not applied	Save sequence (input: FASTA/FASTQ). Smash++ accepts as input FASTA and FASTQ files, in addition to bare sequences. In these cases, the input files are first converted to reads and then processed further. It is possible to save these reads by this option.
`-sp`	N/A<br>Default: not applied	Save profile, *.prf. When the information profile is obtained, Smash++ smoothens then removes it, by default; however, it can be preserved by this option.
`-sf`	N/A<br>Default: not applied	Save filtered file, *.fil. The filtered profile is segmented then removed, by default; however, it can be preserved by this option.
`-ss`	N/A<br>Default: not applied	Save segmented files, *.s_i.
`-sa`	N/A<br>Default: not applied	Save all generated files, including profile, filtered and segmented files.
`-rm`<br>`-tm`	k,[w,d,]ir,α,γ/t,ir,α,γ:...<br>k,[w,d,]ir,α,γ/t,ir,α,γ:...<br>Default:<br>$14, 0, 0.001, 0.95/5, 0, 0.001, 0.95$	Parameters of reference models (for reference-based compression).<br>Parameters of target models (for reference-free compression):<br>• k (integer > 1): context-order size,<br>• w (integer $< 2^{64} - 1$): Count-Min-Log sketch width in $\log_2$ form, e.g., set 10 for $w = 2^{10} = 1024$,<br>• d (integer > 0): Count-Min-Log sketch depth (number of hash functions),<br>• ir (integer: $\{0, 1, 2\}$): inverted repeat, including 0: regular (not inverted), 1: inverted solely, and 2: both regular and inverted,<br>• α (float > 0): estimator,<br>• γ (float: $[0.0, 1.0)$): forgetting factor,<br>• t (integer > 0): threshold (number of substitutions).<br>It is recommended for compression to use "-l" option, since it configures the models automatically. However, using "-rm" and "-tm", one would be able to manually configure the reference and the target models. Parameters of models are described in detail in the section "Methods" of the main paper.
`-ll`	N/A<br>Default: not applied	List of compression levels. It shows the list of parameters that would be chosen automatically for each model.
`-h`	N/A<br>Default: not applied	Usage guide.
`-v`	N/A<br>Default: not applied	More information (verbose).
`--version`		Show the version.

$$
\begin{aligned}
&w[n] = 1, \quad 0 \le n \le N, && \text{(rectangular)}\\
&w[n] = 0.54348 - 0.45652\ \cos\left(\tfrac{2\pi n}{N}\right), \quad 0 \le n \le N && \text{(Hamming)}\\
&w[n] = \sin^2\left(\tfrac{\pi n}{N}\right), \quad 0 \le n \le N && \text{(Hann)}\\
&w[n] = 0.42659 - 0.49656\ \cos\left(\tfrac{2\pi n}{N}\right) + 0.07685\ \cos\left(\tfrac{4\pi n}{N}\right), \quad 0 \le n \le N && \text{(Blackman)}\\
&w[n] = 1 - \left|\tfrac{n-N/2}{N/2}\right|, \quad 0 \le n \le N && \text{(triangular/Bartlett)}\\
&w[n] = 1 - \left(\tfrac{n-N/2}{N/2}\right)^2, \quad 0 \le n \le N && \text{(Welch)}\\
&w[n] = \sin\left(\tfrac{\pi n}{N}\right), \quad 0 \le n \le N && \text{(sine)}\\
&w[n] = 0.35577 - 0.48740\ \cos\left(\tfrac{2\pi n}{N}\right) + 0.14423\ \cos\left(\tfrac{4\pi n}{N}\right) - 0.01260\ \cos\left(\tfrac{6\pi n}{N}\right), \quad 0 \le n \le N && \text{(Nuttall)}
\end{aligned}
$$

(Eq. S1)

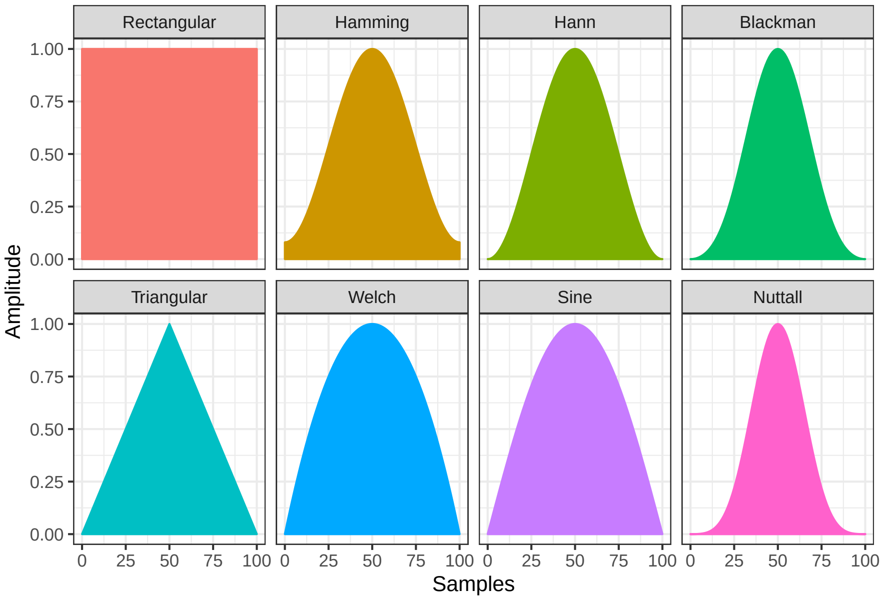

Figure S7. Various windowing functions embedded in Smash++.

By running Smash++, positions of similar regions in reference and target sequences, relative redundancy and also redundancy (complexity) of the regions is saved in a ".pos" file. This tab-delimited file has a header including:

1. The string "##SMASH++" as a specifier for the Smash++ tool,
2. The "PARAM" line to list the parameters used to generate the position file,
3. The "INFO" line to provide the names and sizes of reference and target sequences,
4. The line with the names of columns of the body,

and a body including:

1. RBeg (Reference Begin): beginning of a reference region,
2. REnd (Reference End): end of a reference region,
3. RRelRdn (Reference Relative Redundancy): relative redundancy obtained by compressing the associated target block considering as reference this reference block,

4. RRdn (Reference Redundancy): redundancy (Complexity) of the detected reference block, calculated by reference-free compression,
5. TBeg (Target Begin): beginning of a target region,
6. TEnd (Target End): end of a target region,
7. TRelRdn (Target Relative Redundancy): relative redundancy obtained by compressing the associated reference block considering as reference this target block,
8. TRdn (Target Redundancy): redundancy (Complexity) of the detected target block, calculated by reference-free compression,
9. Inv (Invereted repeat): if the corresponding line is an inverted repeat. "F" (False) means it is regular and "T" (True) means it is inverted.

As an example, the header of the following ".pos" file shows that Smash++ was run by the parameters "-r dataset/REF -t dataset/TAR -l 0 -m 1000" and also the reference "REF" and the target "TAR" are 50,000 base long. The body shows that there is a reference block, from the position 5000 up to 10000, similar to a target block, from the position 2000 to 7000. Relative redundancy of compressing the TAR block, using the REF block as reference, is 1.2473. This number is 1.0187 when the REF block is compressed based on the model of the TAR block. Also, redundancies (complexities) of the REF and the TAR blocks are 1.9010 and 1.8580, respectively. The "F" in the last column shows that this similarity is not of the form inverted repeat. The second record of the body shows an inverted repeat rearrangement.

```
##SMASH++
##PARAM=<-r dataset/REF -t dataset/TAR -l 1 -m 1000>
##INFO=<Ref=REF,RefSize=50000,Tar=TAR,TarSize=50000>
#RBeg  REnd   RRelRdn  RRdn    TBeg   TEnd   TRelRdn  TRdn    Inv
5000   10000  1.0187   1.9010  2000   7000   1.2473   1.8580  F
20000  30000  1.2367   1.9777  40000  30000  1.2545   1.9888  T
```

S1.3 Run visualizer

The position file obtained by Smash++ can be visualized by

```
./smashpp -viz
```

The visualizer provides various options that are described in Table S4. The commands for running the Smash++ visualizer are of the form

```
./smashpp -viz [OPTIONS]  -o <SVG-FILE>  <POS-FILE>
```

Table S4. Options provided by the Smash++ visualizer interface.

Flag	Input	Description
Required		
	*.pos file	Position file, generated by the Smash++ tool. Spaces in the name is supported. It can be redirected to Smash++ by standard input ("stdin").
Optional		
`-o`	*.svg file<br>Default: map.svg	Output image name.
`-rn`	String	Reference name shown on output.
`-tn`	String<br>Default: names in position file's header	Target name shown on output.<br>If it has some spaces, use double quotes, e.g. "Seq label".
`-l`	Integer: $[1, 6]$<br>Default: 1	Type of the link between similar regions.
`-c`	Integer: $[0, 1]$<br>Default: 0	Color mode.

`-p`	Float: $[0.0, 1.0]$<br>Default: 0.9	Opacity.
`-w`	Integer: $[8, 100]$<br>Default: 10	Width of the sequence.
`-s`	Integer: $[5, 200]$<br>Default: 40	Space between sequences.
`-tc`	Integer: $[1, 255]$	Total number of colors in the map. It is automatically chosen by default.
`-rt`<br>`-tt`	Integer: $[1, 2^{32}-1]$<br>Integer: $[1, 2^{32}-1]$	Reference tick size.<br>Target tick size.
`-th`	Integer: $\{0, 1\}$<br>Default: 1	Tick human readable: 0=false, 1=true. If it is true, the sizes on axes are printed in the format 1K, 2M, etc. Note that here, 1K is equivalent to 1000 and not 1024, and so on.
`-m`	Integer: $[1, 2^{32}-1]$<br>Default: 1	Minimum block size. Only the regions with greater sizes than this value will be illustrated.
`-vv`	N/A<br>Default: not applied	Vertical view of the output image.
`-nrr`<br>`-nr`	N/A<br>N/A<br>Default: not applied	Do not show relative redundancy (relative complexity).<br>Do not show redundancy (complexity).<br>Smash++ performs reference-based and reference-free compressions to calculate the NRC and redundancy, respectively. If a user does not tend to show them, he/she can turn them off by "-nrr" and "-nr" triggers.
`-ni`<br>`-ng`	N/A<br>N/A<br>Default: not applied	Do not show inverse maps.<br>Do not show regular (not inverse) maps.<br>Smash++ considers by default both regular and reverse complement maps in its calculations.
`-h`	N/A<br>Default: not applied	Usage guide.
`-v`	N/A<br>Default: not applied	More information (verbose).
`--version`		Show version.

S1.4 Example

This section guides step-by-step employing Smash++ to find and visualize rearrangements between two synthetic sequences. Note that the commands can be run on Linux and macOS, however, they are similar in Windows.

First, install Smash++:

```
git clone https://github.com/smortezah/smashpp.git
cd smashpp
./install.sh
```

Then, copy "smashpp" executable file into "example/" directory and go to that directory:

```
cp smashpp example/
cd example/
```

There is a 1000 byte reference sequence, named "ref", as the following:

```
TCCCGGTCTTTTAGCGGCCAGGGGCCTGGGCTGTATATCGAAAAGTAATATCCCTTTATGCACCGACCGTAATTATGGACAGCAC
ATATACATTATGAGATTTAAAGATCGCGTGGACGACCACGCGGGCTTATAGCCTCACCTGAGGAAGGGGGTGCCTGCGAGGGAGC
TTGAACCTGTAGCCCCAATCTCGAACGACCTGAGGCTTGTGTGGTCAGAGTTGTGACCAGAGCGATCCCGTTGTCAAATCAACCT
AGAGGAGAGGTAAGGGATACGGGTTACATCTCTCCGCTCAGATTGCTCCTATCGGTAGGAAATATCGGGGATAACCCAATACAAA
ACGCTGAACTGTTCATATTTAGCAAGAAGGGGGGACCGAGGAGCTAAATCAGGGACTATGTAAAATTAGAGTTCTAAGGATAGTA
GCACCCGCGCATGGATCGAACTCCGCCTTGGTTGGGTGTGGATGCCATTGGCGTCACCGTGCTGATAGCCAAATCCCTAGTGAAG
GTAACGTGCGGCCAATTAAATAGTAACTGGGCGTAGATTCTAGCGGTGGCCTCATACGGCACCAATGTCCATCCTCCCTGTGCCT
AACACTATAACTCCAATCCCGTGAGTCTACCATCGCCGAGAATCAGACGGGCAACAAACCCAGGGAGCGAGAGCTACGCGGCTTA
TATCACGGATGCTTCCCCACCTAGGGAAATTTGTTCCGCCTAATCCCGTCCTTCGCTGGTCAGGCCCGGTCTAGCGGATTACTTC
```

```
GCCGGTAATGCAGTACAGAATAAATGAAATTCATTAAGAGAATGAAAGGTTATCAGCGAAGCCTTAAGGTCCAACAAGACGTCGA
TATTCTGAAGTAAACTGGTTGACATGTGTGTAACATGAAGCACGCGCTTATTGATATTATCCGCAACCCCACGGCTGGCGGGAAT
CAACCGGCGTCCAGTTCGAACAAGACAGTGCGCTACGCATTACGAAATAGGCTTCGTGTTGCTGT
```

and a 1000 byte target sequence, named “tar”, in this directory:

```
CAGCAACACGAAGCCTATTTCGTAATGCGTAGCGCACTGTCTTGTTCGAACTGGACGCCGGTTGATTCCCGCCAGCCGTGGGGTT
GCGGATAATATCAATAAGCGCGTGCTTCATGTTACACACATGTCAACCAGTTTACTTCAGAATATCGACGTCTTGTTGGACCTTA
AGGCTTCGCTGATAACCTTTCATTCTCTTAATGAATTTCATTTATTCTGTACTGCATTACCGGCGAAGTAATCCGCTAGACCGGG
CCTGACCAGCGAAGGACGGGATTAGGCGGAACAAATTTCCCTAGGTGGGGAAGCATCCGTGATATAAGCCGCGTAGCTCTCGCTC
CCTGGGTTTGTTGCCCGTCTGATTCTCGGCGATGGTAGACTCACGGGATTGGAGTTATAGTGTTAGGCACAGGGAGGATGGACAT
TGGTGCCGTATGAGGCCACCGCTAGAATCTACGCCCAGTTACTATTTAATTGGCCGCACGTTACCTTCACTAGGATCCCGGTCTT
TTAGCGGCCAGGGGCCTGGGCTGTATATCGAAAAGTAATATCCCTTTATGCACCGACCGTAATTATGGACAGCACATATACATTA
TGAGATTTAAAGATCGCGTGGACGACCACGCGGGCTTATAGCCTCACCTGAGGAAGGGGGGGCCTGCGAGGGAGCTTGAACCTGT
AGCCCCAATCTCGAACGACCTGAGGCTTGTGTGGTCAGAGTGGTGACCAGAGCGATCCCGTTGTCAAATCAACCTAGAGGAGAGG
TAAGGGATACGGGTTACATCTCTCCGCTCAGATTGCTCCTATCGGTAGGAAATATCGGGGATAACCCAATACAAAACGCTGAACT
GTTCATATTTAGTAAGAACGGGTGACCGAGGAGCTAAATCAGGGACTATGTAAAATTAGAGATCTAAGGATAGTAGCACCCGCGC
ATGGATCGAACTCCGCCATGGTTTGGTGTCGATGCCATTGGCGTCACCGTGCTGATAGCCAAATC
```

Next, run Smash++ and the visualizer:

```
./smashpp -r ref -t tar
./smashpp -viz -o example.svg ref.tar.pos
```

Results are shown in Fig. S8. Note that the position file is saved as “ref.tar.pos” and the output plot is saved as “example.svg”.

a

```
##SMASH++
##PARAM=<-r ref -t tar>
##INFO=<Ref=ref,RefSize=1000,Tar=tar,TarSize=1000>
#RBeg   REnd    RRelRdn   RRdn     TBeg   TEnd   TRelRdn   TRdn     Inv
0       510     0.5108    2.0000   497    1000   0.5120    2.0000   F
498     1000    0.1518    2.0000   508    0      0.1551    2.0000   T
```

b

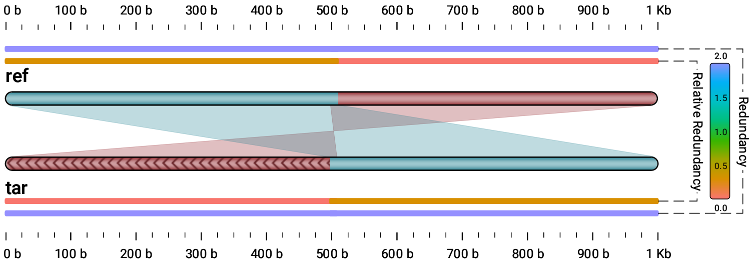

Figure S8. An example of running Smash++ on two 1000 base sequences. (a) the position file and (b) output of the visualizer. One similar region in regular mode and another one in inverted mode are detected.

References

[1] A. E. Darling, B. Mau, and N. T. Perna, "progressivemauve: multiple genome alignment with gene gain, loss and rearrangement," *PloS one*, vol. 5, no. 6, p. e11147, 2010.

[2] Y. Zhang, X. Zhang, T. H. O'Hare, W. S. Payne, J. J. Dong, C. F. Scheuring, M. Zhang, J. J. Huang, M.-K. Lee, M. E. Delany *et al.*, "A comparative physical map reveals the pattern of chromosomal evolution between the turkey (*Meleagris gallopavo*) and chicken (*Gallus gallus*) genomes," *BMC genomics*, vol. 12, no. 1, p. 447, 2011.

[3] J. Lee, W.-y. Hong, M. Cho, M. Sim, D. Lee, Y. Ko, and J. Kim, "Synteny portal: a web-based application portal for synteny block analysis," *Nucleic acids research*, vol. 44, no. W1, pp. W35–W40, 2016.

[4] R. A. Dalloul, J. A. Long, A. V. Zimin, L. Aslam, K. Beal, L. A. Blomberg, P. Bouffard, D. W. Burt, O. Crasta, R. P. Crooijmans *et al.*, "Multi-platform next-generation sequencing of the domestic turkey (*Meleagris gallopavo*): genome assembly and analysis," *PLoS biology*, vol. 8, no. 9, p. e1000475, 2010.

[5] A. U. Sinha and J. Meller, "Cinteny: flexible analysis and visualization of synteny and genome rearrangements in multiple organisms," *BMC bioinformatics*, vol. 8, no. 1, p. 82, 2007.

[6] F. Cabanettes and C. Klopp, "D-GENIES: dot plot large genomes in an interactive, efficient and simple way," *PeerJ*, vol. 6, p. e4958, 2018.

[7] S. L. Salzberg, D. D. Sommer, M. C. Schatz, A. M. Phillippy, P. D. Rabinowicz, S. Tsuge, A. Furutani, H. Ochiai, A. L. Delcher, D. Kelley *et al.*, "Genome sequence and rapid evolution of the rice pathogen *Xanthomonas oryzae* pv. *oryzae* PXO99A," *BMC genomics*, vol. 9, no. 1, p. 204, 2008.

[8] R. Blackman and J. Tukey, "Particular pairs of windows," *The measurement of power spectra, from the point of view of communications engineering*, pp. 95–101, 1959.

[9] J. W. Tukey and R. W. Hamming, *Measuring noise color.* Bell Telephone Laboratories, 1949.

[10] A. Nuttall, "Some windows with very good sidelobe behavior," *IEEE Transactions on Acoustics, Speech, and Signal Processing*, vol. 29, no. 1, pp. 84–91, 1981.

[11] A. V. Oppenheim, R. W. Schafer, and J. R. Buck, *Discrete-Time Signal Processing.* Upper Saddle River, NJ: Prentice Hall, 1999.

[12] F. J. Harris, "On the use of windows for harmonic analysis with the discrete Fourier transform," *Proceedings of the IEEE*, vol. 66, no. 1, pp. 51–83, 1978.

[13] M. S. Bartlett, "Periodogram analysis and continuous spectra," *Biometrika*, vol. 37, no. 1/2, pp. 1–16, 1950.

[14] P. Welch, "The use of fast Fourier transform for the estimation of power spectra: a method based on time averaging over short, modified periodograms," *IEEE Transactions on audio and electroacoustics*, vol. 15, no. 2, pp. 70–73, 1967.
