# Supplementary material for: Smash++: an alignment-free and memory-efficient tool to find genomic rearrangements
Source: Gigascience. 2020 May 20;9(5):giaa048. doi: 10.1093/gigascience/giaa048 (PMC7238676; doi:10.1093/gigascience/giaa048)
Supplement: giaa048_Response_to_Reviewer_Comments_Original_Submission [file giaa048_response_to_reviewer_comments_original_submission.pdf]

Author's Response To Reviewer Comments

Close

Reviewer #1

I found your manuscript overall very well to read. There are some minor typographic mistakes, that I will list consecutivel
below to give you the chance to correct / adjust these to improve the paper.

1. The only comment I have with respect to the paper is that I couldn't find a complete evaluation of the tool with respect
Smash (v1, or the previous version lets say) in terms of computational performance, e.g. runtime/memory requirements f
the mock/synthetic datasets you used to perform the evaluation of Smash++. Also you did some benchmarking in a quali
fashion, showing that Smash++ performs better than Smash, but did not decide to e.g. benchmark other tools (are there
that could be seen as competitor tools/methods.
* We added an experiment to compare quantitatively Smash and Smash++. For such comparison to be fair, Smash++ wa
using only one model (FCM) and also did not compute self-complexity, like Smash. The experiment is described in the sec
"Benchmarking" and the results are shown in Figure 5 and supplementary Table S2.
* As mentioned in the text, since the other tools do not provide relative and self-complexity in addition to detecting simila
we cannot have a fair quantitative comparison with them. In addition, the other methods (excluding Smash) are based on
either alignment or FISH analysis, therefore cannot be seen as "direct competitors" to Smash++, which is a computationa
alignment-free tool.

2. Thank you for creating a bioconda package for the tool, which made installation & usage extremely easy for me. I note
small mistake in the Note S1 - the channel listed for the installation of the method should be bioconda and not your own
cobirna channel to install the tool. This is correct on the Github page, but not on the S1. Thank you also for the really well
Github page where a user can find appropriate information (and I hope a link to the manuscript once its published at som
point) and all the information needed to get to use the method itself - this is not always the case, so it deserves some app
from my side I suppose.
* "cobilab" channel replaced with "bioconda".

3. Typos:
p6, "This can be achieved by "-l 6" option" -> "This can be achieved using the "-l 6" option in Smash++, ...
p10, "is a probabilistic data structure to save frequency" -> " to save the frequency "
* Modified.

==
Reviewer #2

The manuscripts describe a new and improved version of their alignment-free application to find a rearrangements betwe
two FNA sequences. The manuscript describes their improvement using FCM and STMM methods.
The manuscript describes the new version and its application in concise and understandable form, with only minor additio
recommended for the better understanding and for the more practical application of the method.
The visualisation of the two sequences (as per Figures 1, 2,..) would benefit from visualisation in some form the amount o
similarity (I(x)) between the identified regions, to help the user to identify identical repeats compared to similar blocks.

1. The author mentioned the optimisation of the forgetting factor between FCM and STMM, and it relationship with the
complexity. The author might explain this optimisation and if the Kolmogorov complexity is being fed back into this factor.
* We added an experiment to clarify the relation between forgetting factor, context-order size and information content
(complexity). The text in the section "Cooperation of FCMs and STMMs" is accordingly modified to refer to Figure S6, whic
reflects the result of the new experiment.
* The forgetting factor is used to calculate the weights of models (FCMs and STMMs), that are updated for each base as w
move through a sequence. A forgetting factor has a predefined value and is not updated during the calculations; therefore
does not receive any feedback.

2. The authors might elaborate on the parameters used in the reported analysis, giving the users a kind of guideline for se
those parameters.

In this sense, author might explain the reason of using a block size of >100K in most cases and the effect/outcome in usi[illegible] lower block size (for instance on the chicken/turkey or human/chimpanzee data sets). Similarly the use of the threshold between 1.5 and 1.95. In general, is there relationship between the length of the sequences and the 'optimal' parameters used.
* We added some text to the software manual (Note S1) to assist users in setting the parameters.
* On the one hand, if the block size (minimum segment size) is set to a very small value, e.g., 5, Smash++ might detect tiny regions. Choosing a larger value leads to joining of the potential tiny regions. On the other hand, if it is set to a huge value, e.g., 1,000,000,000, many regions (probably all of them) will be discarded. Therefore, a value should be chosen in between by trial and error, with regard to the sequence size. Usually, higher values are more appropriate for larger seque[illegible]
* The threshold is used for segmenting the filtered information content. The more similar the two sequences are, the less entropy is and therefore, a lower threshold can be set. Note that it is not dependant on the sequence sizes.
* In general, some parameters including the ones you mentioned are found by trial and error, and there is no one-size-fit[illegible] solution.

3. The Benchmarking, seems to depend in sequence length for memory and number of similar regions for the CPU wall ti[illegible] The author might comment on 'upper' limits for either of those conditions, and might comment on the code could be parallelised or being distributed on HPC/GPU's.
* The elapsed time depends on both sequence sizes and number of similarities. The memory usage depends mainly on th[illegible] structures used by the models and they depend on the k-mer sizes. As an example, for k=14 a logarithmic table is used a[illegible] data structure, which uses ~1 GB of RAM. This is the case for most of our tests. Note that the space needed for saving th[illegible] mentioned data structure in memory is independent of sequence sizes, since it is basically an array and not a hash table; [illegible] has a fixed size and does not grow. As can be seen in Table S1, larger sequences use only slightly more RAM, which can b[illegible] because of the system I/O buffering operations.
* There is no general upper bound for the time used, since the larger the sequences are and the more regions are detecte[illegible] similar, the more time will be taken. Also, for our whole dataset, the RAM usage is ~1 GB.
* Some parts of the code can be parallelised, such as building the models and compressing different potentially similar re[illegible] The flag "-n" can be used to specify the number of threads for this purpose. Note that the current version of Smash++ is [illegible] specifically tailored to be distributed on HPC environments.

4. Figure 4: Expand the legends as most explanation to the figure is only in the text, such as numbers in the circles,..
* Modified the figure and the associated text.

Close
