# Supplementary material for: Smash++: an alignment-free and memory-efficient tool to find genomic rearrangements
Source: Gigascience. 2020 May 20;9(5):giaa048. doi: 10.1093/gigascience/giaa048 (PMC7238676; doi:10.1093/gigascience/giaa048)
Supplement: giaa048_Response_to_Reviewer_Comments_Revision_1 [file giaa048_response_to_reviewer_comments_revision_1.pdf]

Author's Response To Reviewer Comments

Close

Dear Nicole,

* We added the citation to GigaDB.
* We updated the link to the "dataset" folder in our github repository, in "availability of supporting data and materials" section.
* We added supplementary Figure S6 and supplementary Table S2 to the list of "additional files".
* We updated the "funding" section.

Close
