# Supplementary material for: Smash++: an alignment-free and memory-efficient tool to find genomic rearrangements
Source: Gigascience. 2020 May 20;9(5):giaa048. doi: 10.1093/gigascience/giaa048 (PMC7238676; doi:10.1093/gigascience/giaa048)
Supplement: giaa048_Reviewer_1_Report_Original_Submission — Alexander Peltzer -- 2/12/2020 Reviewed [file giaa048_reviewer_1_report_original_submission.pdf]

Reviewer Report

Title: Smash++: an alignment-free and memory-efficient tool to find genomic rearrangements

Version: Original Submission **Date:** 2/12/2020

Reviewer name: Alexander Peltzer

Reviewer Comments to Author:

Dear authors,
I found your manuscript overall very well to read. There are some minor typographic mistakes, that I will list consecutively below to give you the chance to correct / adjust these to improve the paper.
The only comment I have with respect to the paper is that I couldn't find a complete evaluation of the tool with respect to Smash (v1, or the previous version lets say) in terms of computational performance, e.g. runtime/memory requirements for the mock/synthetic datasets you used to perform the evaluation of Smash++. Also you did some benchmarking in a qualitative fashion, showing that Smash++ performs better than Smash, but did not decide to e.g. benchmark other tools (are there ...?) that could be seen as competitor tools/methods.
Thank you for creating a bioconda package for the tool, which made installation & usage extremely easy for me. I noted a small mistake in the Note S1 - the channel listed for the installation of the method should be bioconda and not your own cobirna channel to install the tool. This is correct on the Github page, but not on the S1. Thank you also for the really well done Github page where a user can find appropriate information (and I hope a link to the manuscript once its published at some point) and all the information needed to get to use the method itself - this is not always the case, so it deserves some applause from my side I suppose.
Typos:
p6, "This can be achieved by "-l 6" option" -> "This can be achieved using the "-l 6" option in Smash++, ..."
p10, "is a probabilistic data structure to save frequency" -> " to save the frequency "
Thank you for making this method available!

Level of Interest

Please indicate how interesting you found the manuscript: Choose an item.

Quality of Written English

Please indicate the quality of language in the manuscript: Choose an item.

Declaration of Competing Interests

Please complete a declaration of competing interests, considering the following questions:

- Have you in the past five years received reimbursements, fees, funding, or salary from an organisation that may in any way gain or lose financially from the publication of this manuscript, either now or in the future?
- Do you hold any stocks or shares in an organisation that may in any way gain or lose financially from the publication of this manuscript, either now or in the future?
- Do you hold or are you currently applying for any patents relating to the content of the manuscript?
- Have you received reimbursements, fees, funding, or salary from an organization that holds or has applied for patents relating to the content of the manuscript?
- Do you have any other financial competing interests?
- Do you have any non-financial competing interests in relation to this paper?

If you can answer no to all of the above, write 'I declare that I have no competing interests' below. If your reply is yes to any, please give details below.

I declare that I have no competing interests.

I agree to the open peer review policy of the journal. I understand that my name will be included on my report to the authors and, if the manuscript is accepted for publication, my named report including any attachments I upload will be posted on the website along with the authors' responses. I agree for my report to be made available under an Open Access Creative Commons CC-BY license (http://creativecommons.org/licenses/by/4.0/). I understand that any comments which I do not wish to be included in my named report can be included as confidential comments to the editors, which will not be published.

Choose an item.

To further support our reviewers, we have joined with Publons, where you can gain additional credit to further highlight your hard work (see: https://publons.com/journal/530/gigascience). On publication of this paper, your review will be automatically added to Publons, you can then choose whether or not to claim your Publons credit. I understand this statement.

Yes Choose an item.
