# Supplementary material for: Smash++: an alignment-free and memory-efficient tool to find genomic rearrangements
Source: Gigascience. 2020 May 20;9(5):giaa048. doi: 10.1093/gigascience/giaa048 (PMC7238676; doi:10.1093/gigascience/giaa048)
Supplement: giaa048_Reviewer_2_Report_Original_Submission — Johannes Zuegg -- 2/23/2020 Reviewed [file giaa048_reviewer_2_report_original_submission.pdf]

Reviewer Report

Title: Smash++: an alignment-free and memory-efficient tool to find genomic rearrangements

Version: Original Submission **Date:** 2/23/2020

Reviewer name: Johannes Zuegg

Reviewer Comments to Author:

The manuscripts describe a new and improved version of their alignment-free application to find a rearrangements between two FNA sequences. The manuscript describes their improvement using FCM and STMM methods.
The manuscript describes the new version and its application in concise and understandable form, with only minor addition recommended for the better understanding and for the more practical application of the method.
The visualisation of the two sequences (as per Figures 1, 2,..) would benefit from visualisation in some form the amount of similarity (I(x)) between the identified regions, to help the user to identify identical repeats compared to similar blocks.
The author mentioned the optimisation of the forgetting factor between FCM and STMM, and it relationship with the complexity. The author might explain this optimisation and if the Kolmogorov complexity is being fed back into this factor.
The authors might elaborate on the parameters used in the reported analysis, giving the users a kind of guideline for setting those parameters.
In this sense, author might explain the reason of using a block size of >100K in most cases and the effect/outcome in using a lower block size (for instance on the chicken/turkey or human/chimpanzee data sets) .
Similarly the use of the threshold between 1.5 and 1.95. In general, is there relationship between the length of the sequences and the 'optimal' parameters to be used.
The Benchmarking, seems to depend in sequence length for memory and number of similar regions for the CPU wall time. The author might comment on 'upper' limits for either of those conditions, and might comment on the code could be parallelised or being distributed on HPC/GPU's.
Figure 4: Expand the legends as most explanation to the figure is only in the text, such as numbers in the circles,..

Level of Interest

Please indicate how interesting you found the manuscript: Choose an item.

Quality of Written English

Please indicate the quality of language in the manuscript: Choose an item.

Declaration of Competing Interests

Please complete a declaration of competing interests, considering the following questions:

- Have you in the past five years received reimbursements, fees, funding, or salary from an organisation that may in any way gain or lose financially from the publication of this manuscript, either now or in the future?
- Do you hold any stocks or shares in an organisation that may in any way gain or lose financially from the publication of this manuscript, either now or in the future?
- Do you hold or are you currently applying for any patents relating to the content of the manuscript?
- Have you received reimbursements, fees, funding, or salary from an organization that holds or has applied for patents relating to the content of the manuscript?
- Do you have any other financial competing interests?
- Do you have any non-financial competing interests in relation to this paper?

If you can answer no to all of the above, write 'I declare that I have no competing interests' below. If your reply is yes to any, please give details below.

None

I agree to the open peer review policy of the journal. I understand that my name will be included on my report to the authors and, if the manuscript is accepted for publication, my named report including any attachments I upload will be posted on the website along with the authors' responses. I agree for my report to be made available under an Open Access Creative Commons CC-BY license (http://creativecommons.org/licenses/by/4.0/). I understand that any comments which I do not wish to be included in my named report can be included as confidential comments to the editors, which will not be published.

Choose an item.

To further support our reviewers, we have joined with Publons, where you can gain additional credit to further highlight your hard work (see: https://publons.com/journal/530/gigascience). On publication of this paper, your review will be automatically added to Publons, you can then choose whether or not to claim your Publons credit. I understand this statement.

Yes Choose an item.
